# Enhanced oncolytic adenoviral production by downregulation of death-domain associated protein and overexpression of precursor terminal protein

**DOI:** 10.1038/s41598-020-79998-1

**Published:** 2021-01-13

**Authors:** Jihyun Lee, Geun-Hyeok Oh, Jeong A. Hong, Soojin Choi, Hye Jin Choi, Jae J. Song

**Affiliations:** 1 Institute for Cancer Research, Yonsei University College of Medicine, Seoul, Republic of Korea; 2 Severance Biomedical Science Institute, Yonsei University College of Medicine, 50-1 Yonsei-ro, Seodaemun-gu, Seoul, Republic of Korea; 3 Department of Internal Medicine, Yonsei University College of Medicine, 50-1 Yonsei-ro, Seodaemun-gu, Seoul, Republic of Korea

**Keywords:** Biochemistry, Cancer, Molecular medicine

## Abstract

Adequate viral replication in tumor cells is the key to improving the anti-cancer effects of oncolytic adenovirus therapy. In this study, we introduced short hairpin RNAs against death-domain associated protein (Daxx), a repressor of adenoviral replication, and precursor terminal protein (pTP), an initiator of adenoviral genome replication, into adenoviral constructs to determine their contributions to viral replication. Both Daxx downregulation and pTP overexpression increased viral production in variety of human cancer cell lines, and the enhanced production of virus progeny resulted in more cell lysis in vitro, and tumor regression in vivo. We confirmed that increased virus production by Daxx silencing, or pTP overexpression, occurred using different mechanisms by analyzing levels of adenoviral protein expression and virus production. Specifically, Daxx downregulation promoted both virus replication and oncolysis in a consecutive manner by optimizing IVa2-based packaging efficiency, while pTP overexpression by increasing both infectious and total virus particles but their contribution to increased viral production may have been damaged to some extent by their another contribution to apoptosis and autophagy. Therefore, introducing both Daxx shRNA and pTP in virotherapy may be a suitable strategy to increase apoptotic tumor-cell death and to overcome poor viral replication, leading to meaningful reductions in tumor growth in vivo.

## Introduction

For many years, oncolytic virotherapy has been a promising anti-cancer gene therapeutic strategy^1^. Oncolytic viruses (OVs) are genetically modified viruses that are able to selectively replicate and produce progeny viruses in cancer cells, resulting in cancer cell lysis^2^. OVs can manipulate the host cellular machinery to create a favorable environment for the production of progeny viruses. Many previous studies have aimed to develop tumor-selective replication systems. E1B55K-deleted adenovirus was engineered to use p53-mediated growth arrest for selectivity in cancer cells, most of which lack functional p53^3^. However, there are some reports that p53 deficiency in cancer cells seems unlikely to be the determinant for oncolytic selectivity of E1B55K-deleted adenovirus, and blocking p53 activity may not be a major requirement for viral replication, or p53 could even promote adenoviral replication^4,5^. Instead, induction of cyclin E, a requirement for adenovirus replication in the absence of E1B55K, could be a molecular marker for oncolytic selectivity and means that E1B55K-like cancer factors are capable of cyclin E induction irrespective of p53 status^6^.

Adenoviral DNA replication is a very efficient process that produces approximately one million copies of viral DNA in infected cells within 40 h^7^. For adenoviral DNA replication, three viral proteins encoded by E2 genes are required: precursor terminal protein (pTP), adenovirus DNA polymerase (Ad Pol), and DNA-binding protein (DBP)^7^. Adenoviral DNA replication starts with protein-primed DNA synthesis initiated by the covalent addition of a dCMP residue to an 80 kDa precursor of pTP^8^. This covalent residue addition to pTP at serine 580 provides a 3′ hydroxyl to stimulate DNA chain extension^9^. The pTP/Ad Pol complex binds to the DNA core region and then pTP-CAT, a pTP-trinucleotide intermediate, jumps back to positions 1–3 at the beginning of the template strand. Shortly after this three-nucleotide jump, Ad Pol dissociates from pTP, leading to progressive DNA elongation. Initiation is enhanced by two cellular factors, nuclear factor I (NFI or CTF1) and nuclear factor III (NFIII or Oct1)^10^. Processing of pTP-DNA creates a distinct template for early transcription (TP-DNA), and serves to guide the template to the appropriate subcellular location throughout the course of infection^11,12^.

For the purpose of viral propagation, several adenoviral gene products and host cellular factors affect or inactivate each other. For example, the early viral gene products E1B55K and E4orf6 associate with host cellular proteins, such as elongin B and C, Cullin5, and Rbx-1, to form the E3 ubiquitin ligase complex that induces proteasomal degradation of target proteins such as p53, integrin α3, MRE11, and RAD50 of the MRE11-RAD50-NBS1 (MRN) complex to prevent both apoptosis prior to virus production, and host defenses^13^. The highly conserved MRN complex is important for sensing, processing, and the repair of double-stranded breaks, and these repair systems counteract DNA virus replication^14^.

A cellular factor known to inhibit the production of adenoviral progeny, Daxx is a multi-functional protein involved in transcriptional regulation, cell apoptosis, disease progression, tumorigenesis, and as an antiviral^15–17^. Daxx is a component of promyelocytic leukemia protein nuclear bodies and a negative regulator of adenovirus serotype-5 replication during productive infection^18–24^. Daxx cooperates with X-linked α-thalassemia retardation syndrome protein (ATRX), a putative member of the sucrose non-fermentable 2 family of ATP-dependent chromatin remodeling proteins^25^. Daxx, in association with ATRX, is involved in histone deacetylase (HDAC) recruitment, H3.3 deposition, and transcriptional repression of cellular promoters. During adenovirus serotype-5 replication, the Daxx/ATRX complex represses virus gene-expression efficiency^25^. In addition to recruiting HDAC in combination with ATRX, Daxx has also been reported to be a transcriptional repressor; it negatively regulated the transcription of a set of autophagy genes known to promote viral adenoviral replication and oncolysis in a time-dependent manner^26–28^.

Here, we develop a way to enhance adenovirus production in many cancer cell types by modulating both viral and cellular factors. In this study, we used E1B55K-deficient oncolytic adenovirus armed with Daxx shRNA, pTP, or both, to compare virus production and tumor-cell death using various human cancer cell lines, and different in vivo mouse models.

## Materials and methods

### Cell lines

The human cancer cell lines DU-145 (human prostate adenocarcinoma), MDA-MB-231 (human breast cancer cell line), U251N (human glioma), PC3 (human prostate adenocarcinoma), A549 (human lung carcinoma), SK-Hep1 (human liver adenocarcinoma), Hep3B (hepatocellular carcinoma), and HPAC (human pancreatic adenocarcinoma), in addition to 293A, a subclone of the human embryonic kidney 293 cell (Invitrogen, Carlsbad, CA, USA), were cultured in Dulbecco’s modified Eagle’s medium with 10% fetal bovine serum (HyClone, Logan, UT, USA). All cells were maintained in a 37 °C humidified atmosphere containing 5% CO_2_.

### Reagents

Antibodies to Daxx, LC3A/B, p53 and acetyl-p53 (Lys382) were purchased from Cell Signaling Technology (Beverly, MA, USA). Antibodies to E1A and β-actin were purchased from Santa Cruz Biotechnology (Santa Cruz Biotechnology Inc, Dallas, TX, USA).

3-MA, rapamycin and pan caspase Inhibitor Z-VAD-FMK were purchased from Promega (Madison, WI, USA). Anti-adenovirus type 5 (Ad5) antibody was purchased from Abcam (Abcam, Cambridge, UK). Antibodies to IVa2, L4-22K, and L4-33K were kindly provided by Dr. Patrick Hearing at SUNY Stony Brook. Restriction enzymes were purchased from New England Biolabs (Ipswich, MA, USA). Trizol was purchased from Life Technologies (Carlsbad, CA, USA), and all other chemicals were purchased from Sigma-Aldrich (St. Louis, MO, USA).

### Polyclonal antibody production of pTP

A 13-mer synthetic peptide, HNRDMTGGVFQLR, was selected as the epitope of pTP from among three candidates^8^. Amino acid sequences were obtained from NCBI (NC_001405.1). Synthetic peptide was immunized into rabbits and serum obtained from the rabbits to make polyclonal antibodies. The overall process, including peptide synthesis, antibody production, and purification, were performed by Bionics (Daegeon, Korea).

### Generation of oncolytic adenovirus expressing Daxx-specific shRNA

The adenoviral shuttle vector, pSP72ΔE3-H1-shDaxx which was previously constructed^23^, was linearized by XmnI digestion. The adenoviral vector dl324-BstBI was linearized by SpeI digestion, and the two linearized vectors were co-transformed into *E. coli* BJ5183 cells for homologous recombination. Then dl324-BstBI-H1-shDaxx after linearization by Bsp1191 was homologously recombined again with adenoviral shuttle vector, pVAX1-3484-CMVp-ΔE1B after linearization by PmeI, which contains an E1 region without E1B55K, to generate E1B55K-deleted oncolytic adenovirus arming Daxx shRNA. The final adenoviral plasmid, dl324-3484-CMVp-ΔE1B-H1-shDaxx, was then digested with PacI and transfected into HEK-293 cells to generate tumor-selective replication-competent adenovirus. The infectious titer of the adenovirus was determined by a limiting dilution assay in 293A cells.

### Generation of pTP-overexpressing oncolytic adenovirus

For cloning the pTP gene of the adenoviral genome into adenoviral shuttle vector, pVAX1-3484-CMVp-ΔE1B, total RNA was isolated from cells with standard Trizol/chloroform extraction following 24 h post infection with E1B55K-deleted oncolytic adenovirus. cDNA was synthesized by RT-PCR using random hexamer. Synthesized cDNA was used as template to perform PCR using primers for the full pTP gene obtained from NCBI (NC_001405.1 or AC_000008). Sense/antisense primer sequences are as follows: 5′-ATGGCCTTGAGCGTCAACGATT-3′, 5′-CTAAAAGCGGTGACGCGGG-3′. PCR products were ligated into pMD20-T vector (Takara, USA), which has a dT-overhang at the 3′ end. Then, PCR product in pMD20-T vector was cloned into the expression vector pCDNA3.1-hygro(+) (Invitrogen, Carlsbad, CA, USA) at the BamHI and XbaI sites. To improve expression of the pTP gene, Kozak sequence (GCCACC) was inserted in front of the pTP gene by annealing. Expression of pTP protein was confirmed by western blotting using pTP antibody. Next, the pTP gene with Kozak sequence was subcloned into an adenoviral shuttle vector, pVAX1-3484-CMVp-ΔE1B, at the HindIII and SalI-blunted sites. The adenoviral shuttle vector, pVAX1-3484-CMVp-ΔE1B-Kozak-pTP, was linearized by PmeI digestion. The adenoviral vector dl324-BstBI was linearized by Bsp119I digestion, and the two linearized vectors were co-transformed into *E. coli* BJ5183 cells for homologous recombination. The following virus amplification step was the same as for other adenoviruses.

### Generation of both Daxx-specific RNA and pTP-expressing oncolytic adenovirus

To generate both Daxx-specific RNA and pTP-expressing oncolytic adenovirus, linearized both pSP72ΔE3-H1-shDaxx shuttle vector by XmnI and dl324-BstBI adenoviral vector by SpeI were co-transformed into *E. coli* BJ5183 cells for homologous recombination. Then dl324-BstBI-H1-shDaxx after linearization by Bsp1191 was homologously recombined again with adenoviral shuttle vector, pVAX1-3484-CMVp-ΔE1B-Kozak-pTP after linearization by PmeI. The final adenoviral plasmid, dl324-3484-CMVp-ΔE1B-Kozak-pTP-H1-shDaxx, was then digested with PacI and transfected into HEK-293 cells to generate tumor-selective replication-competent adenovirus. The following virus amplification step was the same as for other adenoviruses.

### IVa2 cloning

For the cloning of adenoviral IVa2, adenoviral backbone containing IX gene was used as a template for the IVa2 PCR without 5′ end gene (ATGGAAACCAGAG) sense primer 5′-TTATTTAGGGGTTTTGCGCGCG-3′, antisense primer 5′-GGCGAAGACCGGCAGCGCTT-3′ resultant PCR product was inserted into pDrive cloning vector (Qiagen, Hilden, Germany) and selected clones with reverse orientation by sequencing analysis. Then, the annealed DNA fragment designed for the complete construct of IVa2 was added to the HindIII/MscI digested pDrive-IVa2 without 5′ end gene.

The following sequences were prepared for the annealing and adding to IVa2 without 5′ end. Top strand was 5′-AGCTTATGGAAACCAGAGGGCGAAGACCGGCAGCGCTTCAGCACCAGCAGGACCAGCCTCAAGCGCACCCTGG-3′. Bottom strand was 5′-CCAGGGTGCGCTTGAGGCTGGTCCTGCTGGTGCTGAAGCGCTGCCGGTCTTCGCCCTCTGGTTTCCATA-3′. Finally, the cloned IVa2 in pDrive cloning vector was subcloned to HindIII/BamHI-predigested pcDNA3.1 hygro by digestion with HindIII/BamHI for the confirmation of IVa2 expression.

### Titration of virus particles

For calculating the amount of total virus particles, purified virus was dissociated with 0.2% SDS in PBS (pH 7.2) for lysis of viral capsid before titration^29^. Measurements of the optical density (OD) of diluted virus samples at wavelengths 260 and 280 nm using spectrophotometer were performed. For purified virus, the ratio of absorbance at 260 nm and 280 nm should be close to 1.3. The total virus particles were calculated by the following formula:

OD260 reading × dilution factor × 1.1 × 10^12^ particles = number of particles per mL. To calculate the amount of infectious virus particles, viral soup was obtained by freezing and thawing infected cells and supernatant three times followed by centrifugation to obtain the pellet. Viral soup or purified virus was serially diluted in the range of 10^−2^–10^−12^ and each 100 μl of dilutes were added to a 1 × 10^4^ 293A packaging cell. After 7 days of incubation at 37 °C, the number of wells showing cytopathic effect (CPE) in 293A were counted. The amounts of infectious virus particles were then calculated by the following formula:

Titer (TCID50/mL) = 10^(1 + 1 (counted wells − 0.5) TCID50/mL, to transform TCID50/ml in PFU/ml, Titer (PFU/ml) = 1 × 10^(TCID50/mL-0.7).

### Calculation of the number of viral DNA copies

Following infection of same amount of each virus, cultured cell and media were harvested after 24 h of infection. Then, viral DNA after CsCl density gradient ultracentrifugation and Tris-dialysis of harvested viral soup was purified by Exgene Viral DNA/RNA kit (GeneAll, Seoul, Korea) as provided in protocol, and then measured absorbance at OD260 and OD280. Copy numbers were calculated by the formula which requires amount of DNA and length of template considering molecular weight. (The formula used is: number of copies = (ng * 6.022 × 10^23^)/(length * 1 × 10^9^ * 650)).

### Oncolytic assay for cytopathic effect (CPE)

To compare the CPE of replication-competent adenoviruses (Ad-3484-shNC, Ad-3484-shDaxx), cells were first plated to ~ 80% confluency into 24-well plates. They were infected with various multiplicities of infection (MOIs) of each adenovirus. After 48–72 h of infection depending on the time point of complete lysis at 0.1 MOI, the remaining cells on the plate were fixed with 4% paraformaldehyde for 30 min and stained with 0.05% crystal violet for 10 min.

### Immunoblot analysis

Cells were lysed in 1× Laemmli lysis buffer (62.5 mM Tris, pH 6.8, 2% sodium dodecyl sulfate, 10% glycerol, 0.02% bromophenol blue). Proteins were separated by SDS-PAGE and electrotransferred onto polyvinylidene fluoride (PVDF) membranes (Millipore, Billerica, MA, USA). After blocking each membrane (5% non-fat dry milk in TBS–Tween-20 (0.1%, v/v) at room temperature for 1 h, the membrane was then incubated with the primary antibody (diluted according to the manufacturer's instructions) for 2 h ~ overnight depending on the antibody. Horseradish peroxidase-conjugated anti-mouse or anti-rabbit or IgG was used as a secondary antibody. Immunoreactive proteins were visualized by chemiluminescence (ECL; Thermo Fisher Scientific, Waltham, MA, USA). The images of the original blots are available within supplementary information file. Many original blots were cut prior to hybridization with antibodies to have the same experimental condition as much as possible and for convenience’ sake if the molecular size of expected proteins are clearly different, for example Daxx and caspase 8.

### Immunohistochemistry (IHC)

For IHC, tumor tissues of two mice per group after 7 days of third virus injection were extracted after CO_2_ euthanasia. Then, they were fixed for 24 h in 10% formaldehyde, and embedded in paraffin. Tissue sections were deparaffinized twice with xylene for 10 min and were rehydrated using a graded alcohol series. After antigen retrieval, the sections were permeabilized with 0.5% PBX (0.5% Triton X-100 in PBS) for 30 min and washed three times with PBS. After blocking for 1 h with 5% BSA, the sections with primary antibodies were incubated overnight at 4 °C. Primary Antibody Enhancer (Thermo Fisher Scientific) and horseradish peroxidase Polymer (Thermo Scientific) were used for signal amplification. To develop the colored product, a mixture of DAB (3,3′-diaminobenzidine) Plus Chromogen and DAB Plus Substrate (Thermo Fisher Scientific) was added for 5 min. After washing with PBS, 20% hematoxylin counterstain was added for 2–5 min to stain the nuclei. After dehydration and clearing twice in xylene, the tissue sections were coverslipped with mounting media (xylene:mount = 1:1) for microscopy.

### Animal study

Animal study was carried out in compliance with the ARRIVE guidelines as follows. Animal studies were conducted following approved protocol (2018-0138) established by Yonsei University Health System’s Institutional Animal Care and Use Committee (IACUC) in accordance with the guidelines of the Animal Welfare Act and the Guide for the Care and Use of Laboratory Animals. After receiving the approval, eighty 5-week-old male BALB/c athymic nude mice, weighing around 20 g, were obtained from OrientBio (Seongnam, Korea). All mice were housed for 1 week for a period of adjustment, and free feeding to food (PicoLab Rodent diet 20 5053) and purified water by reverse osmosis in individually ventilated cages were provided at a temperature of 21 ± 0.2 °C, humidity of 50 ± 10%, and a 12/12-h light/dark cycle. The maximum caging density was five mice and aspen chip after autoclave was used as bedding. To generate a xenograft tumor model, each of 1 × ${10}^{7}$ A549 or DU145 cancer cells were injected with Matrigel into the subcutaneous abdominal region of mice. When the tumors reached an average size of 100–120 mm^3^, 1 × 10^9^ plaque forming unit (PFU) of each adenoviruses diluted in 50 μl PBS or PBS alone was intratumorally injected into the nude mice. Each mice group with similar average and standard deviation values of tumor size was separated (n = 7 per group). The adenoviruses injected were Ad-3484-NC, Ad-3484-shDaxx, Ad-3484-pTP, Ad-3484-pTP-shDaxx. Intratumoral injection was repeated every other day for a total of three injections. Five mice per group were used for the tumor size measurement, and two mice per group were used for the immunohistochemistry. Regression of tumor growth was assessed by measuring the length (L) and width (W) of tumors. Tumor volume was calculated using the following formula: Volume (mm^3^) = 0.52 × L × W^2^.

### Statistical analysis

Data are presented as the mean ± standard error of mean (S.E.M.). Differences between groups were examined using unpaired two-tailed t tests. *P* values were calculated using GraphPad Prism version 6.0 (Systat Software Inc.). *P* < 0.001, *P* < 0.01 or *P* < 0.05 was considered statistically significant. All experiments were performed three times independently.

## Results

**Figure 1:**
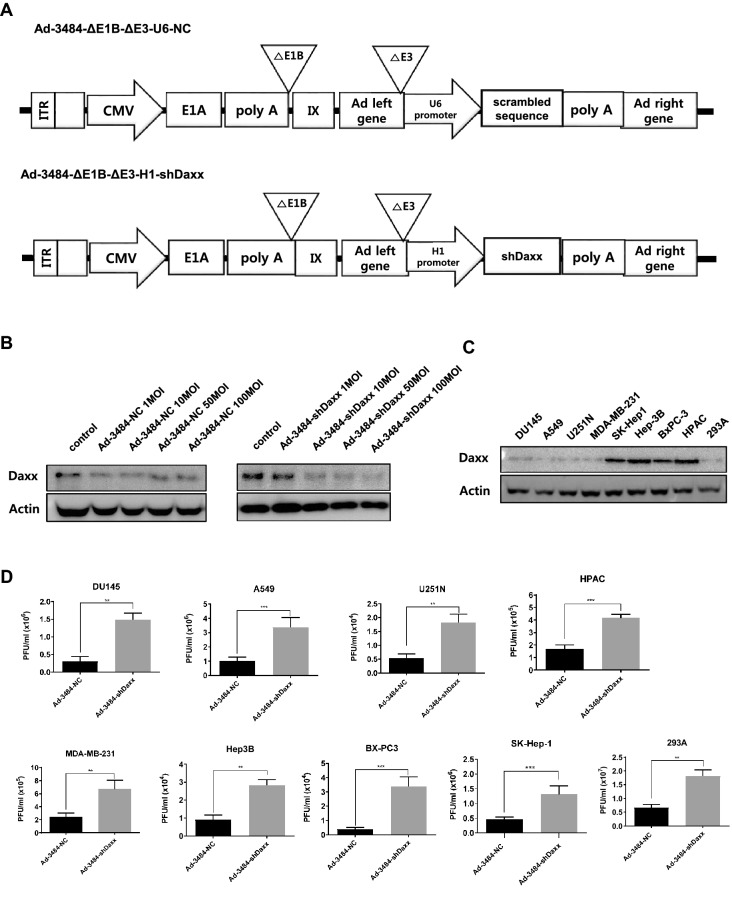

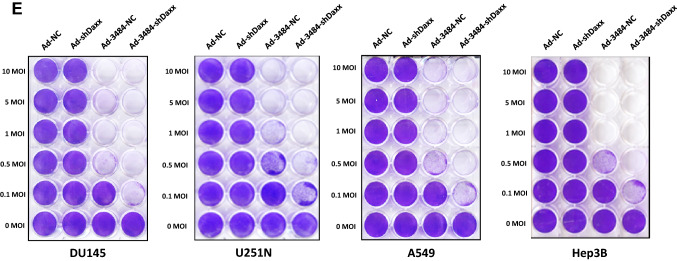
Enhanced adenovirus production by oncolytic adenovirus armed with Daxx shRNA. (**A**) Scheme of E1B55K-deleted oncolytic adenovirus armed with scrambled RNA (Ad-3484-NC) or Daxx shRNA (Ad-3484-shDaxx). (**B**) Expression of Daxx protein after infection with Ad-3484-NC or Ad-3484-shDaxx at the indicated MOIs for 48 h in DU145 cells. (**C**) Endogenous Daxx protein levels in various human cancer cells. (**D**) Plaque-forming unit (PFU) titration assays of adenovirus produced by each human cancer cell line following Ad-3484-NC or Ad-3484-shDaxx infection at MOI 50 for 48 h with exchanged media after PBS washing. Error bars represent standard errors from three independent experiments. *P* values less than 0.05 were considered statistically significant (*P < 0.05; **P < 0.01; ***P < 0.001). (**E**) Crystal violet oncolytic assay of DU145 (left), U251N (middle), and A549 cells (right) following Ad-NC, Ad-shDaxx, Ad-3484-NC, or Ad-3484-shDaxx infections at MOIs between 0 and 10 for 72 h. All cells remaining on plates were fixed with 4% paraformaldehyde and stained with 0.05% crystal violet.

Daxx has been previously reported to be a negative regulator of adenovirus replication, suggesting that adenovirus has evolved to mediate proteasomal degradation of Daxx protein using viral proteins such as E1B55K and E4orf6^18,19^. Although E1B55K-deleted oncolytic adenovirus, which selectively replicates in cancer cells that have mutant or p53-null status, is useful in terms of selectivity, it cannot induce proteasomal degradation of Daxx. Therefore, Daxx levels in cancer cells remain high after infection by E1B55K-deleted oncolytic adenovirus. Downregulation of Daxx could be helpful to increase viral production, we therefore constructed E1B55K-deleted oncolytic adenovirus armed with Daxx shRNA (Ad-3484-shDaxx). To confirm that Daxx was downregulated, levels of Daxx protein were assessed following infection either by Ad-3484-shDaxx or E1B55K-deleted oncolytic adenovirus armed with scrambled RNA (Ad-3484-NC) (Fig. 1A). Daxx protein levels decreased depending on Ad-3484-shDaxx multiplicity of infection (MOI), but not for Ad-3484-NC (Fig. 1B), implying that sequence-specific Daxx downregulation occurred. Cellular Daxx protein showed various levels of expression in human cancer cells (Fig. 1C). To quantify infectious virus particles produced by Ad-3484-shDaxx compared to Ad-3484-NC, a 50% tissue culture infective dose (TCID50) titration assay was performed using viral soup obtained from both cancer cell lines and the 293 adenovirus packaging cell line infected with 50 MOI (0.1 MOI for 293 cells) Ad-3484-shDaxx and Ad-3484-NC, respectively. In all of the cancer cell lines examined, titrations showed significant increases (2.5–7.5 fold) in virus production for Ad-3484-shDaxx compared to Ad-3484-NC, and this viral production range was not related to p53 status. For example, A549 and SK-Hep1 cells (wild-type p53) produced relatively higher amount of virus, whereas Hep3B and BX-PC3 cells (mutant p53) produced relatively lower amounts of virus (Fig. 1D). Next, oncolytic assays were performed to confirm whether the increased production of virus progeny by Ad-3484-shDaxx was the main contributor to increased cell lysis in the various cancer cells rather than the cellular effects of Daxx downregulation. Increased cell death was observed with Ad-3484-shDaxx virus at 1 MOI, but not with the replication-deficient Ad-shDaxx (Fig. 1E). These results demonstrated that increased cell lysis was caused by greater virus production, not by the cellular effects of Daxx downregulation.

### Daxx downregulation increased infectious virus particle rather than total virus particles

**Figure 2:**
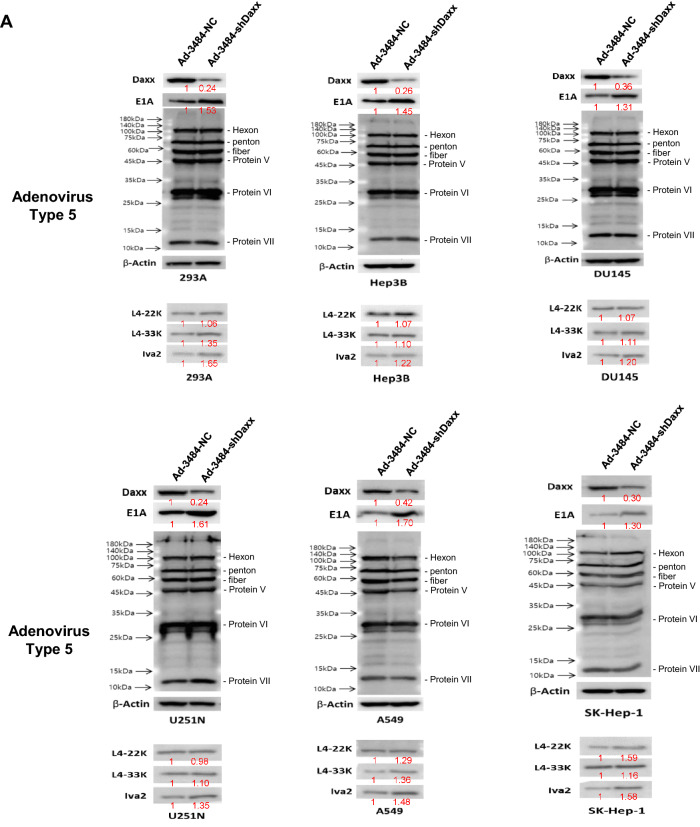

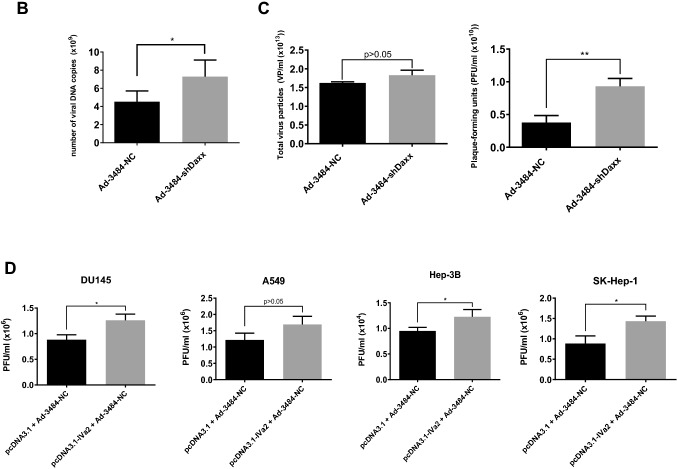
Increased viral infectivity by Daxx downregulation. (**A**) Western blot analysis for the detection of viral late gene products was performed in a variety of cancer cells as well as 293A cells infected with Ad-3484-NC or Ad-3484-shDaxx at 50 MOI for 48 h. The numbers indicate the relative band intensity of Ad-3484-shDaxx to control (Ad-3484-NC) after band intensities were measured with a densitometer. (**B**) Estimation of viral DNA copy number after infection with Ad-3484-shDaxx at 10 MOI for 24 h in 293A cells. After infection for 24 h, cultured cells and media were harvested. Purified DNA (CsCl gradient and Tris-dialysis of virus soup) was measured at OD260 and OD280 for copy-number calculations. Error bars represent standard errors from three independent experiments. *P* values less than 0.05 were considered statistically significant (*P < 0.05). (**C**) Virus particle estimation. After CsCl gradient and Tris-dialysis purification of the virus soup from 293A cells following infection with the same amount of each oncolytic adenovirus (Ad-3484-NC and Ad-3484-shDaxx), total virus particles (VP/ml) were calculated by measuring absorbance at OD260 (left) and the amounts of infectious virus particles were successfully calculated by TCID50 titration assays in 293A cells using plaque-forming units (PFU/ml) (right). Error bars represent standard errors from three independent experiments. *P* values less than 0.05 were considered statistically significant (*P < 0.05). (**D**) Infectious viral particles were estimated after transfection of pcDNA3.1-IVa2 followed by oncolytic control adenovirus in various HCC. Error bars represent standard errors from three independent experiments. *P* values less than 0.05 were considered statistically significant (*P < 0.05).

Adenoviral protein levels after viral infection in various cell lines were investigated after Daxx downregulation. Unexpectedly, adenoviral protein levels of capsid proteins (e.g., Hexon, Penton, protein V, and VI) and protein VII did not differ significantly between cells infected with Ad-3484-NC or Ad-3484-shDaxx (Fig. 2A). Although these detected proteins (adenoviral late-gene transcription products) showed no clear differences, levels of E1A protein (an early-gene transcription product involved in viral replication) and IVa2 protein (known to be among the late-gene transcription products L422K, L433K, and IVa2 involved in viral DNA packaging^30–33^) increased following Ad-3484-shDaxx infection in the various cells (Fig. 2A). Taken together, these results indicate an increase in gene copies by Daxx downregulation (Fig. 2B), and an increase in infectious viral particles without increasing adenoviral protein levels or total virus particles with Ad-3484-shDaxx compared to Ad-3484-NC amplified from 293A cells (Fig. 2C). Next, we tested the possibility of increased viral packaging via increased IVa2 levels induced by Daxx downregulation. To test this, we used exogenous IVa2 gene expression in various hepatocellular carcinoma followed by oncolytic control adenovirus (Ad-3484-NC) challenge. Figure 2D shows that infectious viral particles were significantly increased by exogenous IVa2 expression.

### pTP increased progeny virus production

**Figure 3:**
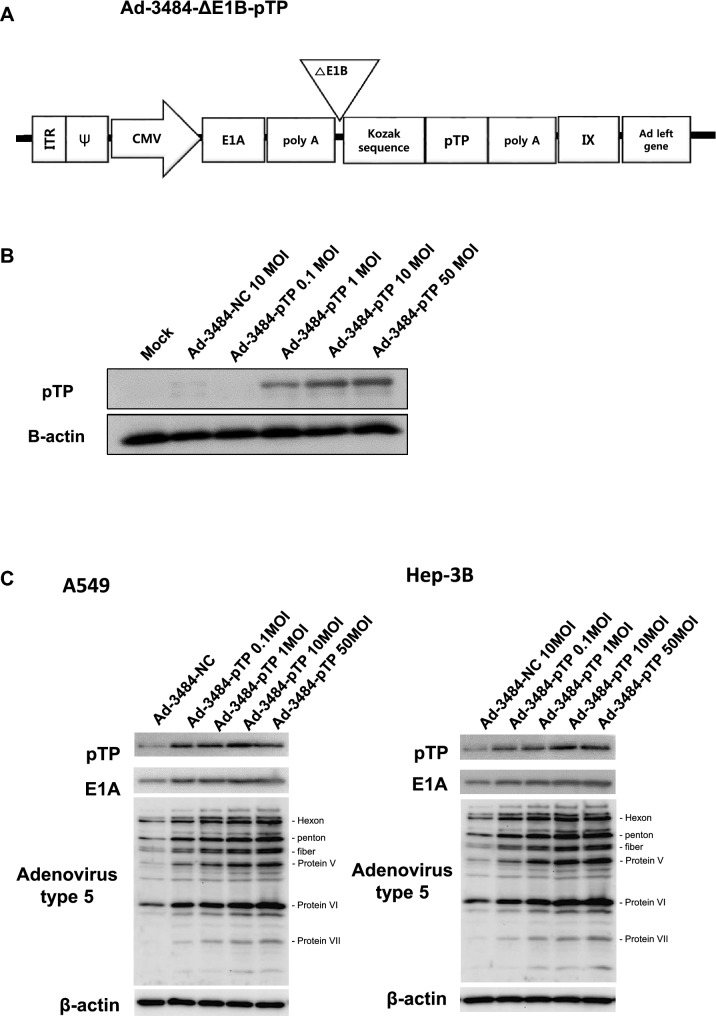

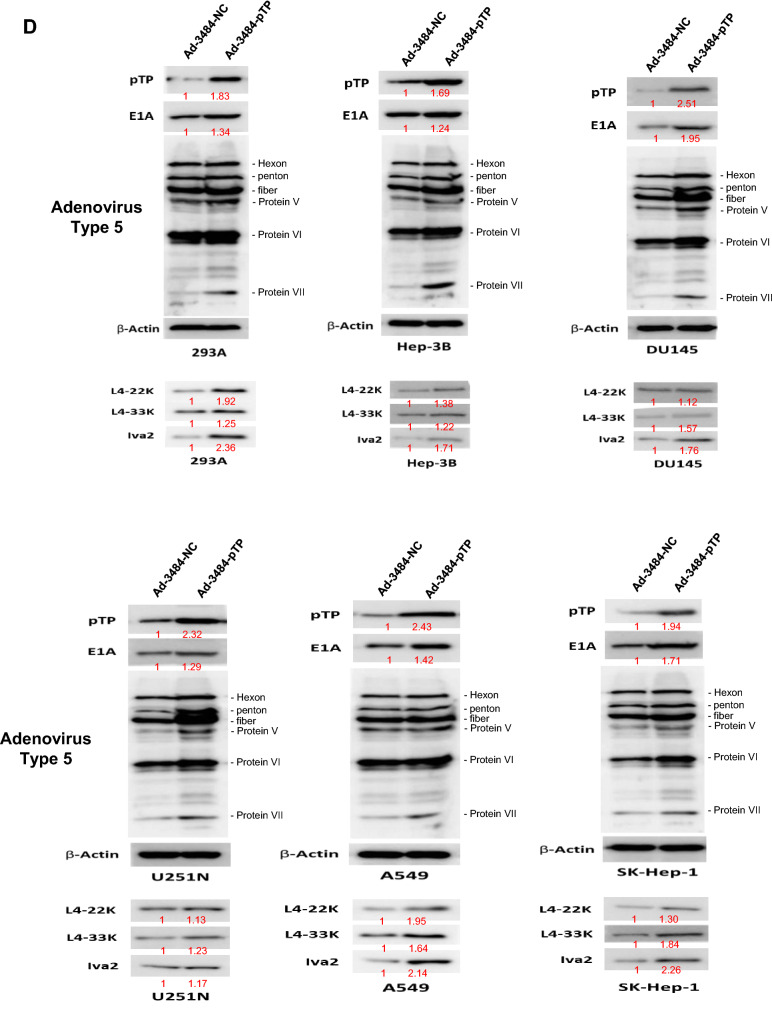

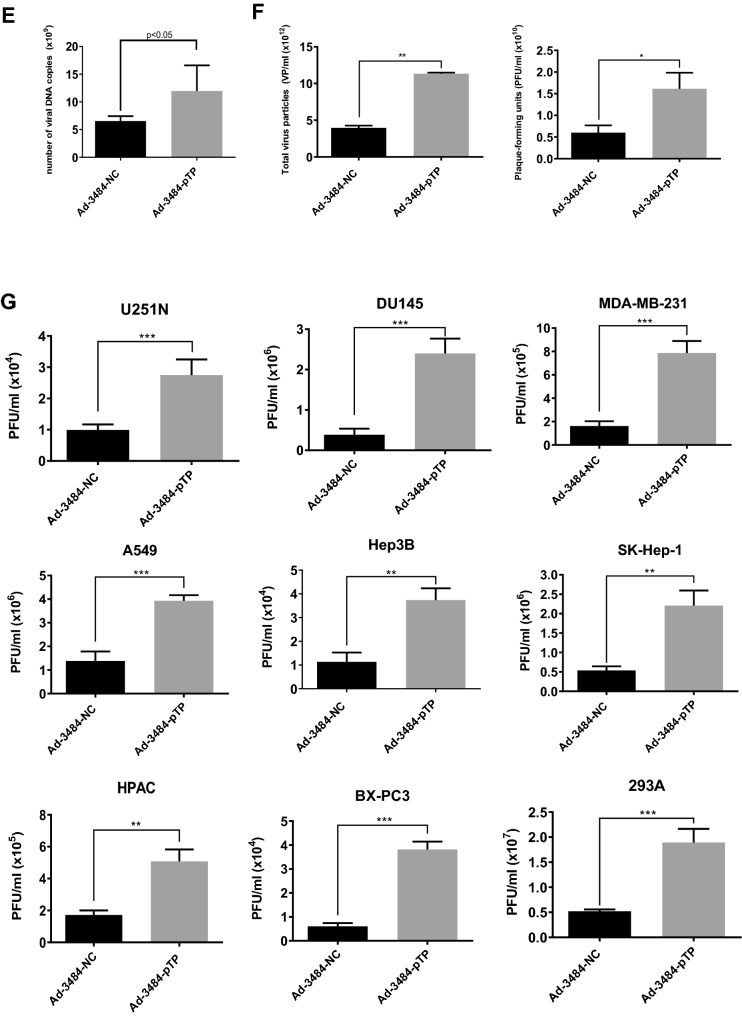
Enhanced production of progeny virus by introduction of the precursor terminal protein (pTP) gene. (**A**) Scheme of pTP-overexpressing E1B55K-deleted oncolytic adenovirus. (**B**) Expressions of pTP protein in DU145 cells after Ad-3484-pTP infection at indicated MOIs for 48 h. (**C**) Western blot analysis for detection of protein levels, including Adenovirus type 5, in A549 and Hep3B cells infected using various MOIs of Ad-3484-pTP for 48 h. (**D**) Western blot analysis for detection of protein levels, including adenovirus type 5, in various cancer cells as well as in 293A cells infected with 10 MOIs of Ad-3484-pTP for 48 h. The numbers indicate the relative band intensity of Ad-3484-pTP to control (Ad-3484-NC) after band intensities were measured with a densitometer. (**E**) Estimation of viral DNA copy number after pTP overexpression. After infection with Ad-3484-pTP at 10 MOI for 24 h in 293A cells, cells and media were harvested. Purified DNA after CsCl density gradient ultracentrifugation and Tris-dialysis of viral soup was measured at OD260 and OD280 for copy-number calculations. Error bars represent standard errors from three independent experiments. (**F**) After infections with the same amount of each oncolytic adenovirus (MOI 10 with Ad-3484-NC or Ad-3484-pTP), and purification of virus following CsCl density gradient ultracentrifugation and Tris-dialysis of viral soup from 293A cells, total virus particles (VP/ml) were calculated by measuring absorbance at OD260 and plaque-forming units (PFU/ml), which indicates that the amounts of infectious virus particles were successfully calculated by TCID50 titration assays in 293A cells. Error bars represent standard errors from three independent experiments. *P* values less than 0.05 were considered statistically significant (*P < 0.05). (**G**) PFU titration assays of adenovirus produced by each human cancer cell line following Ad-3484-NC or Ad-3484-pTP infections at MOI 50 for 4 h, and incubations for 48 h with media exchanged after PBS washing. Adenovirus packaging cells (293A) were infected at MOI 0.1. Error bars represent standard errors from three independent experiments. *P* values less than 0.05 were considered statistically significant (**P < 0.01; ***P < 0.001).

Even though Daxx downregulation increased infectious viral particle, it was important to assess other factor(s) that could enhance adenovirus replication, in order to maximize its oncolysis efficacy. Experiments designed to increase the expression of pTP, a protein required for initiating viral gene replication into adenovirus structures^7^, were then performed. pTP has been shown to promote post-infection initiation of viral gene replication by binding with cellular factors NFI and Oct-1^7–10,34^. E1B55K-deleted oncolytic adenovirus constructed with the full gene coding for pTP (Ad-3484-pTP) was generated (Fig. 3A), and pTP expression was confirmed using an antibody that recognized the pTP epitope HNRDMTGGVFQLR (Fig. 3B). pTP overexpression led to increased E1A expression in various cancer cells (Fig. 3C,D), as well as increases in other viral proteins, including viral genome packaging-related proteins. As a result, viral genome increases and increases in total virus particles were seen, concomitant with increased infectious viral particle (Fig. 3E,F). Using a variety of cancer cells, this pTP overexpression resulted in increased infectious viral particle approximately 2.5–6 fold (Fig. 3G); more infectious viral particle in most of the examined cancer cell types than that seen with Ad-3484-shDaxx. As demonstrated in Fig. 3D, the expression of IVa2 (induced by pTP overexpression) increased more compared to other packaging-related proteins (L422K and L433K) and may have also increased packaging efficiency.

### Both pTP overexpression and Daxx downregulation significantly increased. infectious viral particle

**Figure 4:**
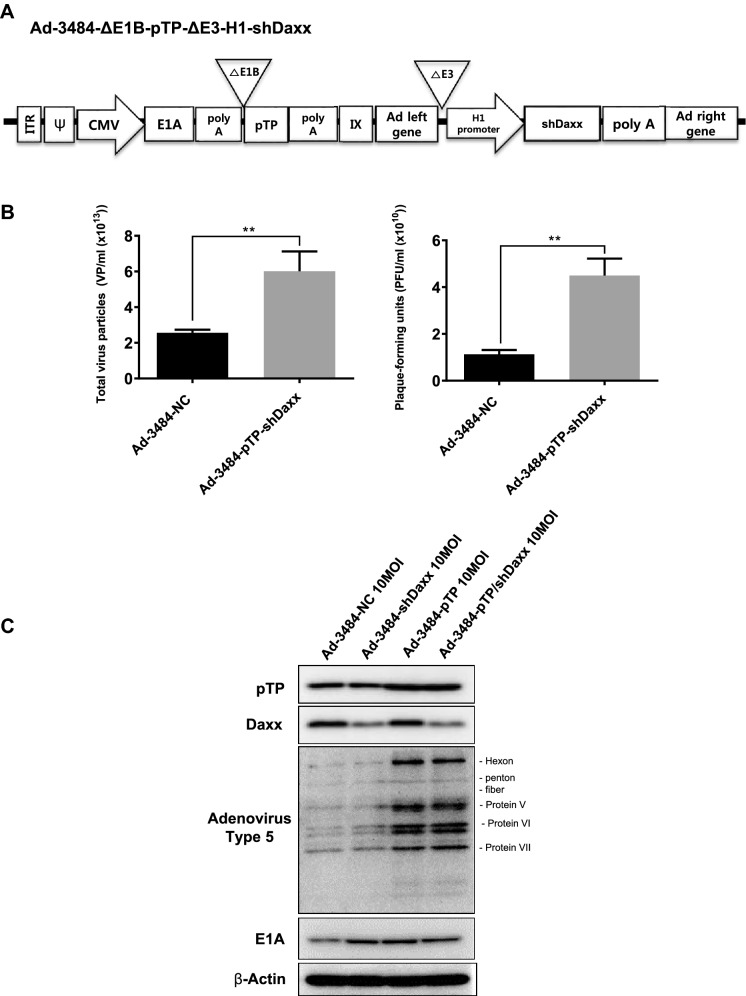

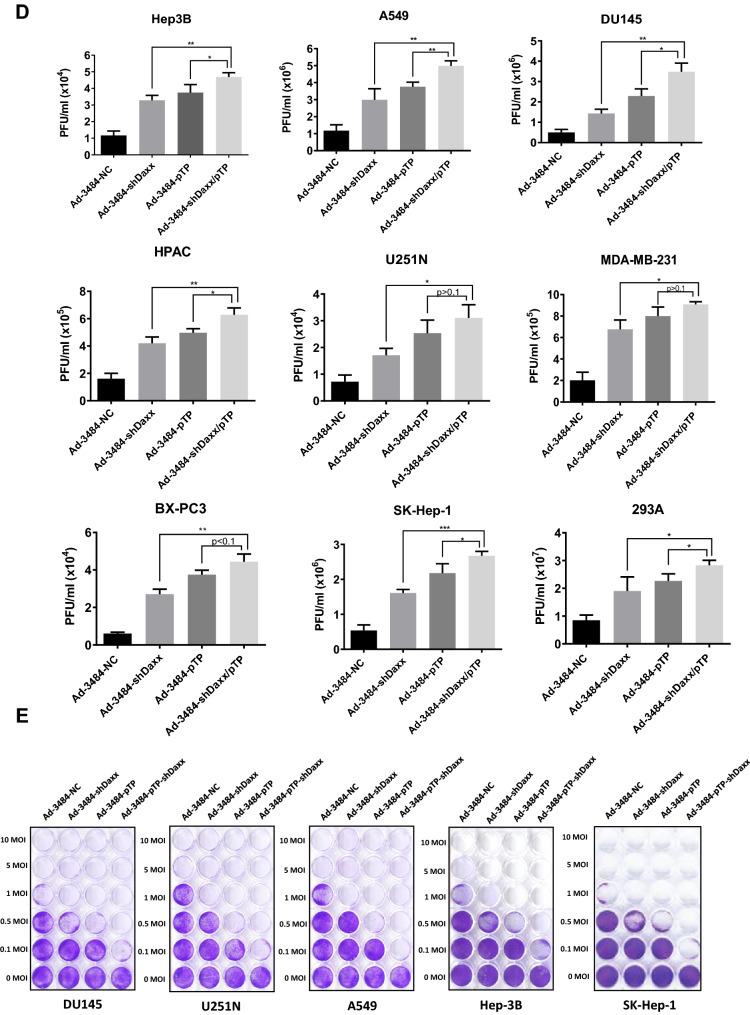

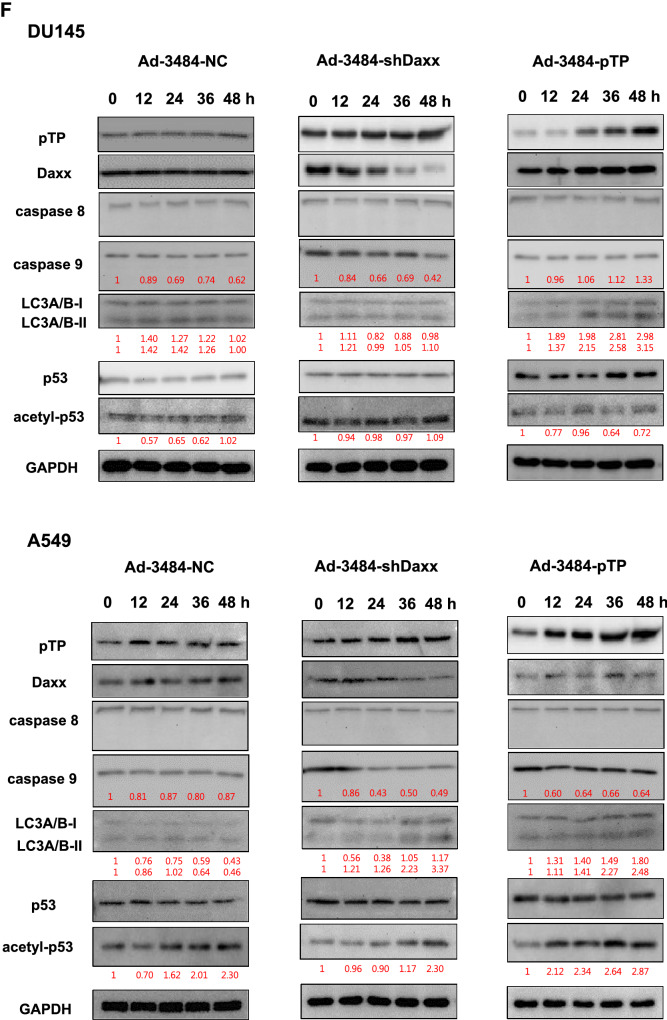

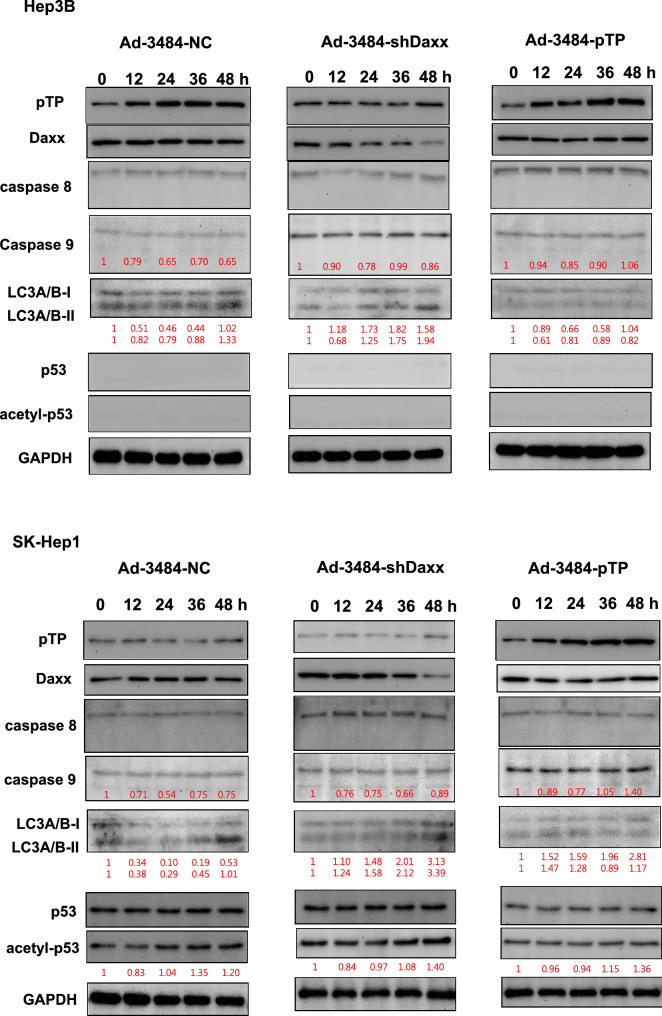

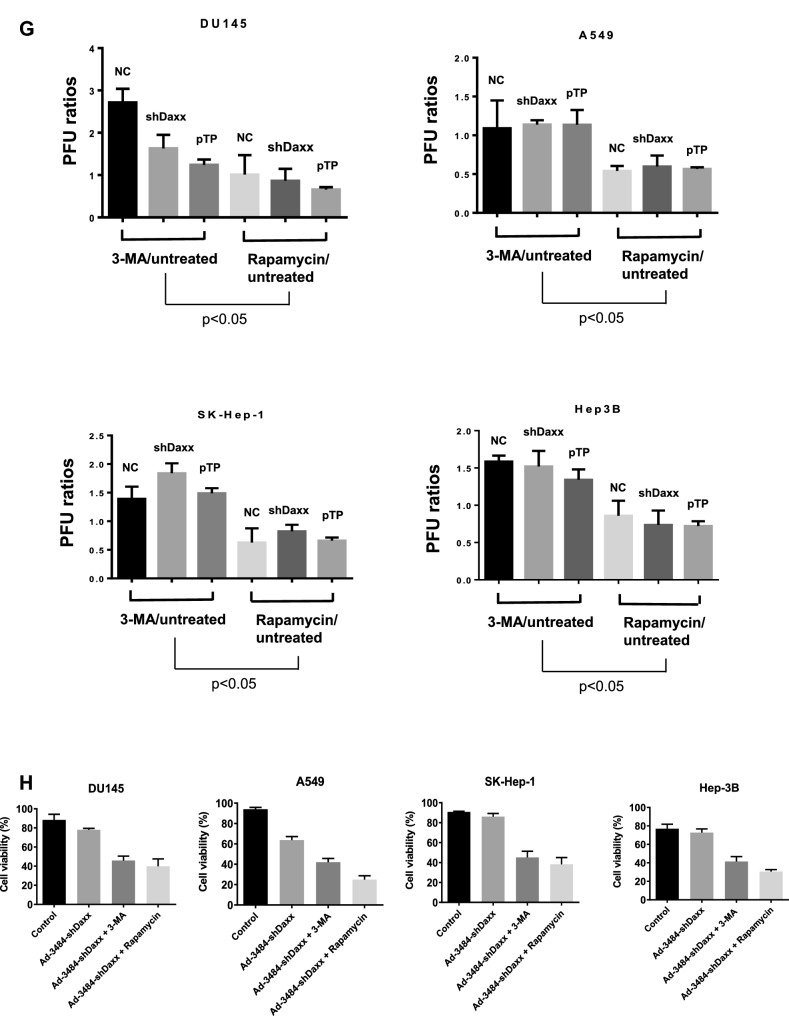

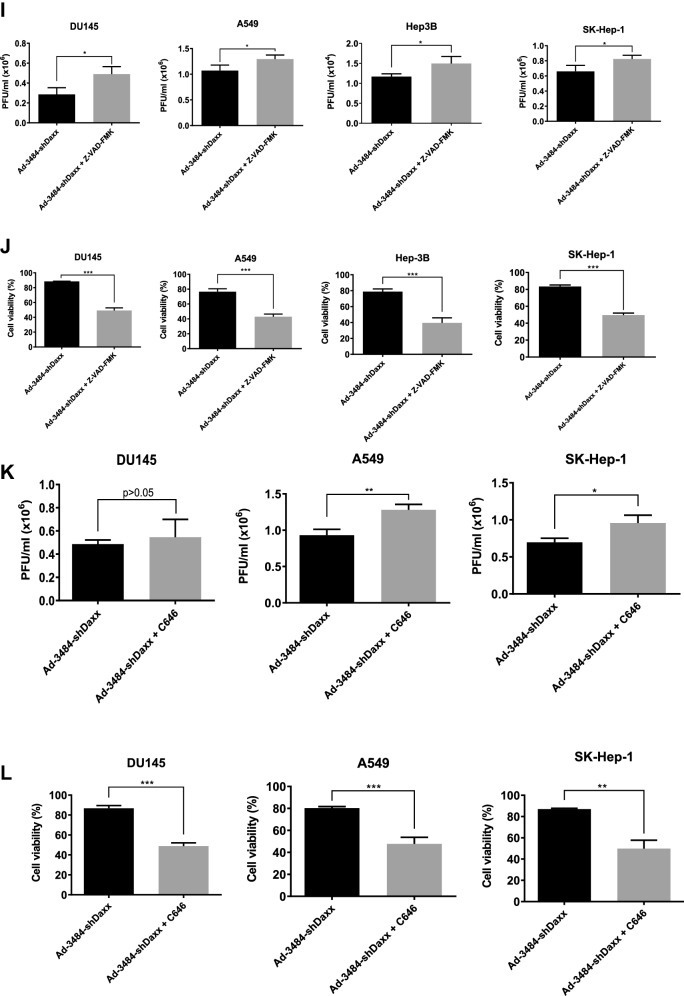
Enhanced virus production by both pTP and Daxx shRNA expressing E1B55K-deleted oncolytic adenovirus (Ad-3484-pTP-shDaxx). (**A**) Scheme of pTP and Daxx shRNA-both expressing E1B55K-deleted oncolytic adenovirus. (**B**) Virus particle amounts were determined after purification through a CsCl gradient and Tris-dialysis of virus soup from 293A cells. Following infection with the same amount of infectious oncolytic adenovirus (Ad-3484-NC and Ad-3484-pTP-shDaxx), total virus particles (VP/ml) (left) and plaque-forming units (PFU/ml) (right) were calculated. Error bars represent standard errors from three independent experiments. *P* values less than 0.05 were considered statistically significant (**P < 0.01). (**C**) Western blot analysis for the detection of protein levels, including adenovirus type 5, from DU145 cells infected with 10 MOI of Ad-3484-NC, Ad-3484-shDaxx, Ad-3484-pTP, or Ad-3484-pTP-shDaxx for 48 h. (**D**) PFU titration assays of adenovirus produced by each human cancer cell line following Ad-3484-NC, Ad-3484-shDaxx, Ad-3484-pTP, or Ad-3484-pTP-shDaxx infections at MOI 50 for 4 h, and incubation for 48 h with media exchanged after PBS washing. Adenovirus packaging cells (293A) were infected at MOI 0.1. Error bars represent standard errors from three independent experiments. (**E**) Oncolytic assays of various cancer cells (DU145, U251N, A549, Hep3B, and SK-Hep1) infected with Ad-3484-NC, Ad-3484-shDaxx, Ad-3484-pTP, or Ad-3484-pTP-shDaxx at MOIs between 0 and 10, for 72 h. All cells remaining on the plates were fixed with 4% paraformaldehyde and stained with 0.05% crystal violet. (**F**) Western blot analysis for detection of caspase 8, 9, LC3A/B-I, II, p53 and acetylated p53 (Lys382) protein levels in various cancer cells infected with 10 MOIs of Ad-3484-NC or Ad-3484-shDaxx or Ad-3484-pTP for each indicated time. The numbers indicate the relative band intensity of infected samples for each indicated time to control (0 h) after band intensities were measured with a densitometer. (**G**) Plaque-forming unit (PFU) titration assays of adenovirus in various cancer cells treated with autophagy inhibitor (3-MA 10 mM) or autophagy enhancer (rapamycin 50 nM) for 44 h after 4 h of infections with 10 MOIs of Ad-3484-NC, Ad-3484-shDaxx, or Ad-3484-pTP. PFU ratios mean that viral titer of 3-MA or rapamycin-treated cancer cells after infection NC, shDaxx or pTP virus divided by viral titer of untreated cancer cells after infection NC, shDaxx or pTP virus respectively. Error bars represent standard errors from three independent experiments. NC, Ad-3484-NC; shDaxx, Ad-3484-shDaxx; pTP, Ad-3484-pTP (**H**) Cell viability in various cancer cells treated with autophagy inhibitor (3-MA 10 mM) or autophagy enhancer (rapamycin 50 nM) for 44 h after 4 h of infections with 10 MOIs of Ad-3484-shDaxx. Cell viability was estimated by trypan blue exclusion. Error bars represent standard errors from three independent experiments. (**I**) Plaque-forming unit (PFU) titration assays of adenovirus in various cancer cells treated with pan caspase inhibitor (Z-VAD-FMK 10 μM) for 44 h after 4 h of infections with 10 MOIs of Ad-3484-shDaxx Error bars represent standard errors from three independent experiments. *P* values less than 0.05 were considered statistically significant (*P < 0.05). (**J**) Cell viability in various cancer cells treated with pan caspase inhibitor (Z-VAD-FMK 10 μM) for 44 h after 4 h of infections with 10 MOIs of Ad-3484-shDaxx. Cell viability was estimated by trypan blue exclusion. Error bars represent standard errors from three independent experiments. *P* values less than 0.05 were considered statistically significant (***P < 0.001). (**K**) Plaque-forming unit (PFU) titration assays of adenovirus in various cancer cells treated with histone acetyltransferase inhibitor (C646 25 μM) for 44 h after 4 h of infections with 10 MOIs of Ad-3484-shDaxx. Error bars represent standard errors from three independent experiments. *P* values less than 0.05 were considered statistically significant (*P < 0.05; **P < 0.01). (L) Cell viability in DU145, A549 and SK-Hep1 treated with histone acetyltransferase inhibitor (C646 25 μM) for 44 h after 4 h of infections with 10 MOIs of Ad-3484-shDaxx. Cell viability was estimated by trypan blue exclusion. Error bars represent standard errors from three independent experiments. *P* values less than 0.05 were considered statistically significant (***P < 0.001).

We next examined the effects of combining pTP overexpression with Daxx downregulation on virus production in vitro. E1B55K-deleted oncolytic adenovirus expressing both pTP and Daxx shRNA (Ad-3484-pTP-shDaxx) was generated (Fig. 4A), and pTP expression and Daxx downregulation were confirmed. Total viral particle and infectious viral particle were compared between Ad-3484-NC and Ad-3484-pTP-shDaxx, and both were found to be significantly increased with pTP expression and with Daxx downregulation (Fig. 4B), while the expression pattern of viral proteins, including E1A, was almost the same with pTP expression only (Fig. 4C). Overall, infectious viral particle increased in the order of shDaxx < pTP < shDaxx/pTP in the various cancer cells examined (Fig. 4D), resulting in enhanced tumor-cell lysis (Fig. 4E). We also investigated whether viral production was preceded by apoptotic death or autophagic death, and the timing of autophagy/apoptosis induced by shDaxx or pTP from various cancer cells (Fig. 4F). Minor autophagy or apoptosis occurred after Daxx downregulation or pTP overexpression, depending on the p53-related cancer cell types. This minor autophagy likely originated from our own viral structure of E1B-deleted oncolytic adenovirus. Piya et al. also reported that E1B19K was linked to beclin 1-mediated autophagic cell death^28,35^. This inhibited or enhanced autophagy, compared to untreated infections, also showed a tendency for increased or decreased infectious viral particle production, respectively (Fig. 4G), and a negative correlation between the induction of autophagic cell death and virus replication, a contradictory result to Rodriguez-Rocha et al.^28^. Unexpectedly, cell viability was also decreased even after treatment with autophagy inhibitor (3-MA) (Fig. 4H), suggesting either enhanced cell death due to increased late viral lysis due to blocked autophagy, or enhanced cell death due to increased autophagy by rapamycin. Curiously, increased infectious viral particle production and decreased cell viability were also observed even in 3-MA-treated DU145 cells showing no LC3-II increase after Daxx downregulation (Fig. 4F–H), suggesting the possibility of LC3-independent autophagy^36^. In addition, caspase-dependent apoptosis also had the tendency to inhibit viral production by shDaxx regardless of p53 status (Fig. 4I). Intriguingly, cell death was enhanced even under conditions of caspase inhibition, probably due to increased late viral lysis (Fig. 4J). On the other hand, wild-type p53 acetylation greatly increased with both Daxx downregulation (A549, SK-Hep1) and pTP overexpression (A549) (Fig. 4F). Here, p53 acetylation was used as a biomarker for p53 activation leading to apoptosis, as well as cell-cycle arrest^37,38^. C646, a histone acetyltransferase p300 inhibitor acting as a p53 activation inhibitor, significantly increased viral production in p53 wild-type cancer cells (Fig. 4K), and decreased cell viability (Fig. 4L), suggesting that inhibition of p53 activation also enhanced cell death derived from viral lysis. Interestingly, decreased cell viability also occurred after treatment with a p53 acetylation inhibitor in p53 mutant DU145 cells (for reference, Hep3B cells are p53 null) which showed no increase of p53 acetylation with Daxx downregulation (Fig. 4F), possibly through p300 inhibition acting like an inducer of apoptosis in prostate cancer cells^39^.

### Both pTP overexpression and Daxx downregulation effectively suppressed tumor growth

**Figure 5:**
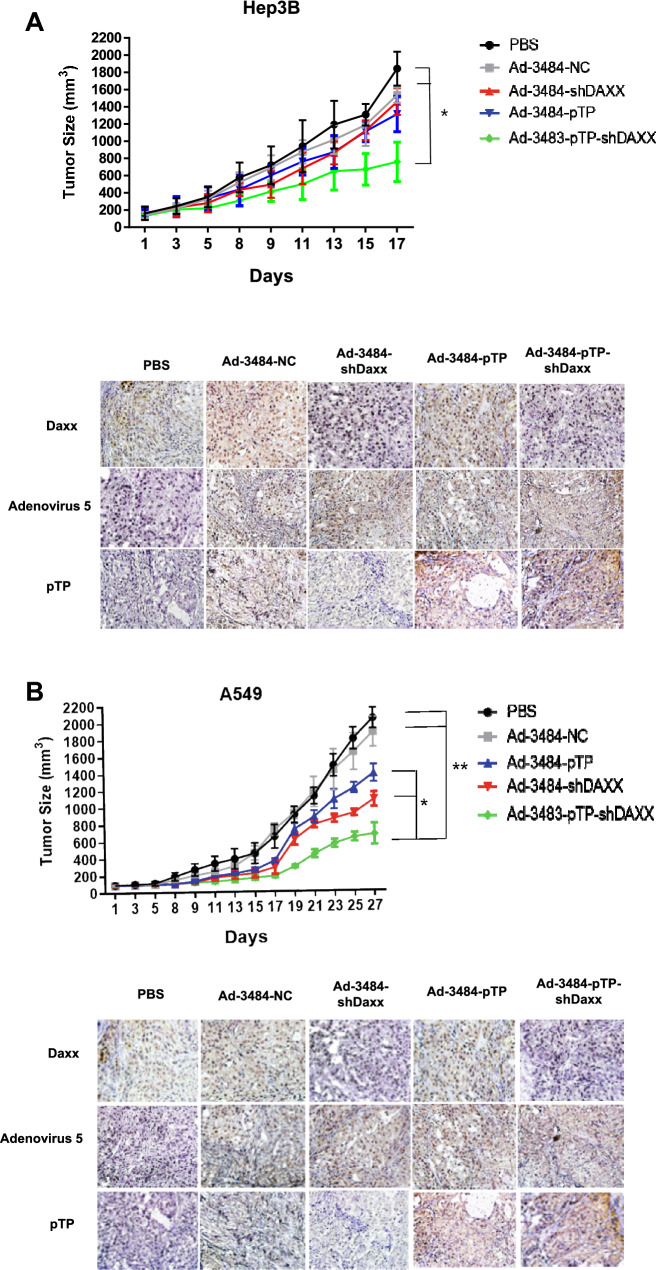
Tumor size measurements and immunohistochemical images of tumors. Hep3B cells (**A**) or A549 cells (**B**) in Matrigel were injected subcutaneously in the abdominal region of BALB/c athymic nude mice, and intratumoral injections of PBS, or of each virus indicated, were repeated every other day for a total of three injections. Tumor volume was measured and calculated on the indicated days using the following formula: Volume (mm^3^) = 0.52 × (Length) × (Width)^2^ (n = 5 per group) (upper). Error bars represent standard error from five mice in each experimental group. The asterisk indicates a significant difference between each comparative group (*P < 0.05; **P < 0.01). Tumor tissues after euthanasia from each group (n = 2 per group) for immunohistochemistry were harvested 7 days following virus infections. Adenovirus 5, pTP or Daxx primary antibodies were incubated overnight at 4 °C on tissue slides. Images were obtained by light microscopy (200×). (lower).

**Figure 6:**
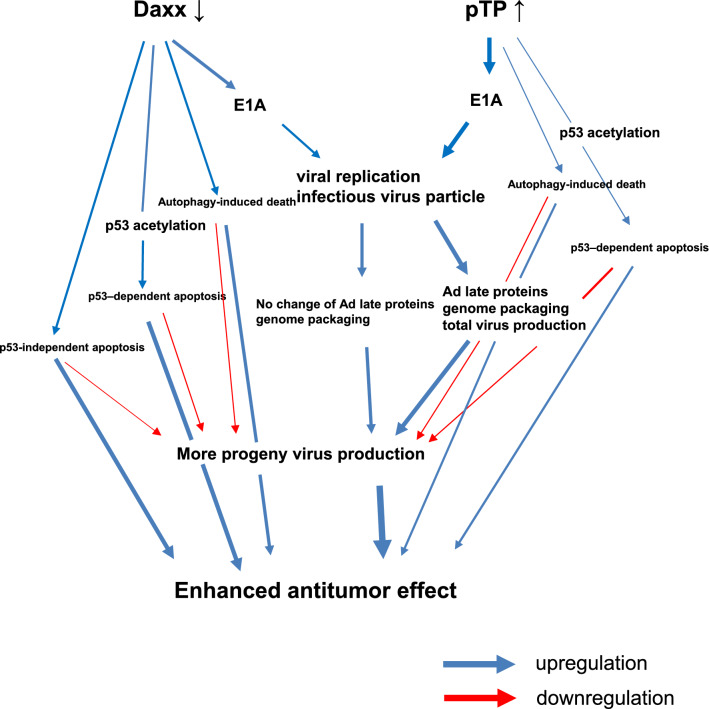
Schematic diagram of enhanced viral production and anti-tumoral effect by a combination of Daxx downregulation, and pTP upregulation, in E1B55K/19K deleted oncolytic adenovirus. pTP upregulation greatly increased viral production, while Daxx downregulation increased mainly infectivity, but not total virus particles. Additionally, Daxx downregulation induced stronger apoptotic cell death than pTP expression did in tumor cells with wild type p53, resulting in an enhanced anti-tumoral effect. This suggests that the combination of both Daxx downregulation and pTP upregulation in oncolytic adenovirus therapy could be an ideal cooperative pairing.

To confirm the antitumor effects of increasing virus production by pTP overexpression and Daxx downregulation, we performed in vivo experiments in xenograft animal models using Hep3B and A549 cell lines. Effective tumor suppression was observed in the Ad-3484-pTP-shDaxx group compared to the Ad-3484-NC group, suggesting that efficient virus production resulted in tumor regression in vivo (Fig. 5A,B). The immunohistochemical data suggested that the combination of pTP and Daxx downregulation was more effective for virus replication than in the single groups (Ad-3484-pTP or Ad-3484-shDaxx), leading to enhanced tumor suppression. Interestingly, tumor regression was greater in A549, a p53 wild-type tumor after shDaxx infections than after pTP infections, suggesting that apoptotic death (caspase activation + p53 activation) as well as autophagy induction were more effective in diminishing tumor sizes in functional p53 tumor types despite sacrificing viral production. In summary, Fig. 6 provides a schematic diagram of enhanced viral production and anti-tumoral effect by a combination of Daxx downregulation, and pTP upregulation, in E1B55K/19K deleted oncolytic adenovirus.

## Discussion

In this study, we have shown enhanced virus production by silencing Daxx, by overexpressing pTP, or in combination. To improve the efficacy of oncolytic adenovirus therapy in cancer patients, adequate virus replication and production of infectious progeny virus for cell lysis (oncolysis) in tumors, are indispensable. In previous clinical trials and in vivo tests, most viruses deteriorated in tumors, resulting in failures to reduce tumor mass. There were several reasons for these: limited virus delivery to adjacent tumor cells, despite reaching the tumor site, because of abundant extracellular matrix and stromal cells; insufficient virus replication in tumor cells; high interstitial fluid pressure; and host antiviral defense systems^40–43^. Notwithstanding changes to current OV therapy to include antitumor immune-modulating agents, attention to the prerequisites for virus production could be still one of the most effective ways to improve antitumor efficacy^40^. Here, we studied the control of cellular- and/or viral-factor expression related to virus replication for the purpose of overcoming insufficient virus production, and investigating the different mechanisms of viral production by Daxx downregulation and pTP expression. Daxx, a repressor of virus replication, remains at high levels in human cancer cells after E1B55K-deleted adenovirus infection, because viral protein E1B55K, the major component of E3 ligase complex with E4orf6, is required for proteasomal degradation of Daxx protein^18^. Schreiner et al*.* explained the mechanism by which Daxx protein represses viral replication with ATRX^25^. Blocking Daxx/ATRX complex assembly, by E1B55K-mediated proteasomal degradation of Daxx protein, may be required for the efficient transcription of adenoviral genes. We previously demonstrated that silencing Daxx encouraged adenovirus early-gene transcription that was required for viral DNA replication^7^, but this silencing of Daxx was not sufficient to increase intermediate-early or late viral gene transcription products. In addition to enhance direct viral replication, Daxx downregulation was found to promote both autophagy and apoptosis^17,26^. Here, we investigated the possibility that both autophagy and apoptosis were indispensable for efficient virus production. Autophagy may generate nutrients that can be used for producing viral progeny^28^, and apoptotic cell death plays a central role in the timely release of progeny virus from infected cells, but also in host defenses by blocking viral expansion at initial stages of infection^44^. It is reasonable to suppose that many viruses have evolved mechanisms to either inhibit or activate host-cell death depending on their needs. This means that, depending on the circumstances, inhibition of apoptosis may sometimes allow viruses to optimize replication and progeny synthesis by prolonging the infected cell's life, otherwise host-cell apoptosis would release progeny virions. For example, maximal virus production is very dependent on timing; earlier cell death inhibited sufficient viral production, while later cell death induced adequate viral release. For our experimental conditions, Daxx downregulation and/or pTP overexpression increased viral production during various types of cancer-cell death and decreased viral production efficiency to some extent (Fig. 4).

In addition, for the E1B55K-deficient shDaxx downregulation experiments, p53 functionality (A549 cells with wild-type p53) was necessary for efficient tumor regression, supporting the view that shDaxx downregulation with functional p53 enhances apoptosis, promotes adenoviral replication, and increases late viral gene expression^5^ rather than the competing view that successful adenovirus replication requires inactivation of p53. The present results have also shown that the timing of cellular apoptosis and autophagy are very important for adequate viral production; late death for sufficient viral release, followed by tumor regression. pTP overexpression enhanced overall viral replication irrespective of p53 status, while shDaxx had a tendency to increase both viral production in p53-mutant cancer cells, and apoptotic cell death in p53 wild-type cancer cells. The appearance of cell death that accompanied Daxx downregulation and/or pTP overexpression enhanced viral production is likely a negative byproduct of, rather than a promoter of, viral production, and maximal viral release might be achieved only by viral E4orf4-mediated apoptosis^45^. Overall, it is more reasonable to regard the early activation of various types of cell death during virus replication simply as a host defense mechanism for combating viral infection^46^. Thus, the timing of various types of cell death induced by viral oncolytic replication needs further investigation. pTP upregulation greatly increased viral production, while Daxx downregulation mainly increased infectivity, but not total virus particles. In addition, Daxx downregulation induced a stronger apoptotic cell death response in tumor cells with wild-type p53 than pTP expression did, resulting in an enhanced anti-tumoral effect. This suggests that Daxx downregulation combined with pTP upregulation in oncolytic adenovirus therapy could be an ideal cooperative pairing. Therefore, the combination of shDaxx and pTP could provide a very satisfactory antitumor influence on a variety of tumor types with different genetic backgrounds.

## Supplementary Information

Supplementary Information.

## Abbreviations

PBS: Phosphate buffered saline
shhDaxx: ShRNA of human Daxx
shmDaxx: ShRNA of mouse Daxx
RT-PCR: Reverse-transcriptional PCR
SDS–PAGE: Sodium dodecyl sulfate–polyacrylamide gel electrophoresis
