# Supplementary material for: Enhanced oncolytic adenoviral production by downregulation of death-domain associated protein and overexpression of precursor terminal protein
Source: Sci Rep. 2021 Jan 13;11:856. doi: 10.1038/s41598-020-79998-1 (PMC7807022; doi:10.1038/s41598-020-79998-1)
Supplement: Supplementary file 1 — Supplementary Information. [file 41598_2020_79998_MOESM1_ESM.pptx]

## Slide 1
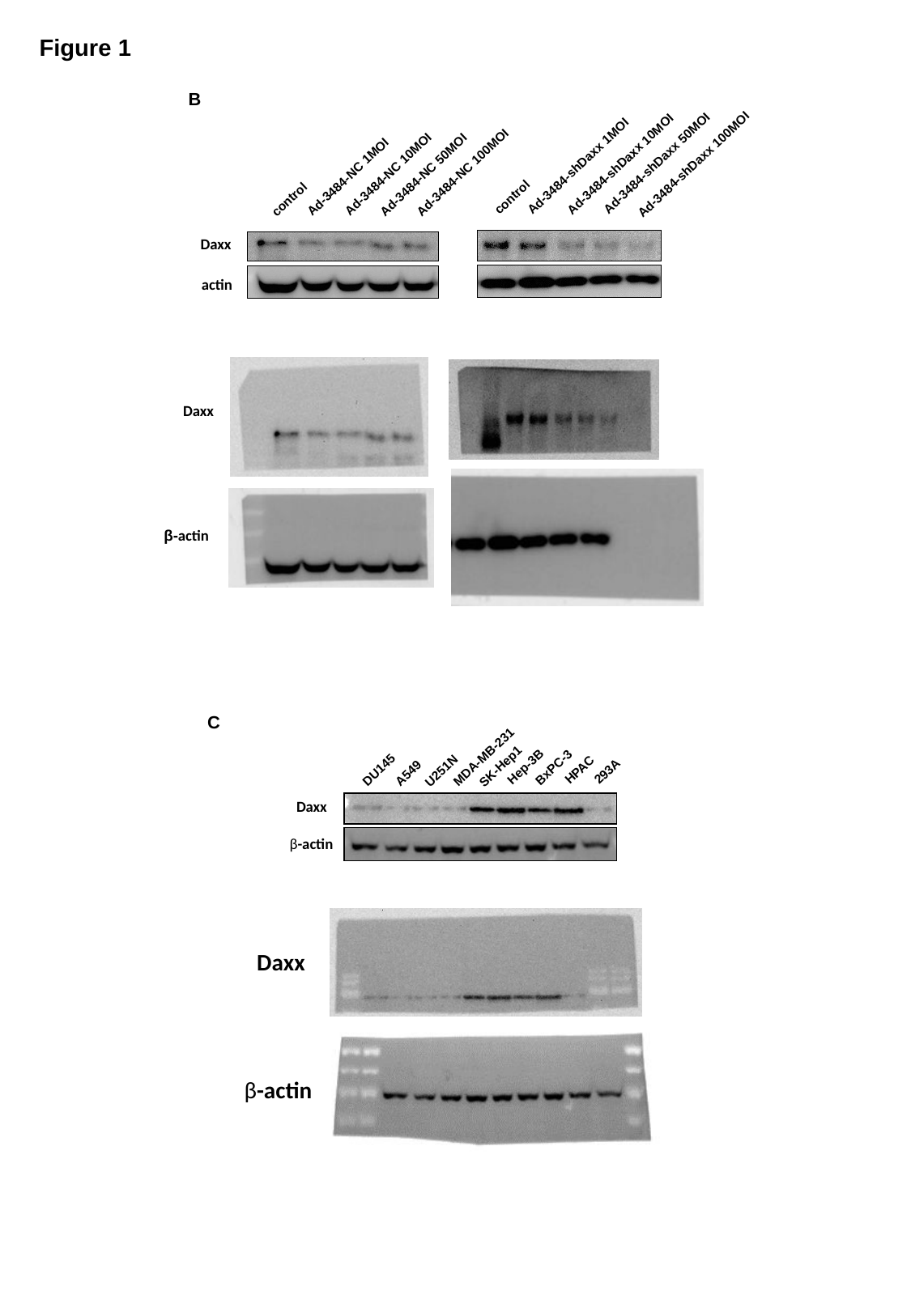

Figure 1
B
Ad-3484-shDaxx 1MOI
Ad-3484-shDaxx 50MOI
Ad-3484-shDaxx 10MOI
Ad-3484-shDaxx 100MOI
Ad-3484-NC 100MOI
Ad-3484-NC 50MOI
Ad-3484-NC 10MOI
Ad-3484-NC 1MOI
control
control
Daxx
actin
Daxx
β-actin
C
HPAC
Hep-3B
BxPC-3
A549
DU145
MDA-MB-231
SK-Hep1
U251N
293A
Daxx
β-actin
Daxx
β-actin

## Slide 2
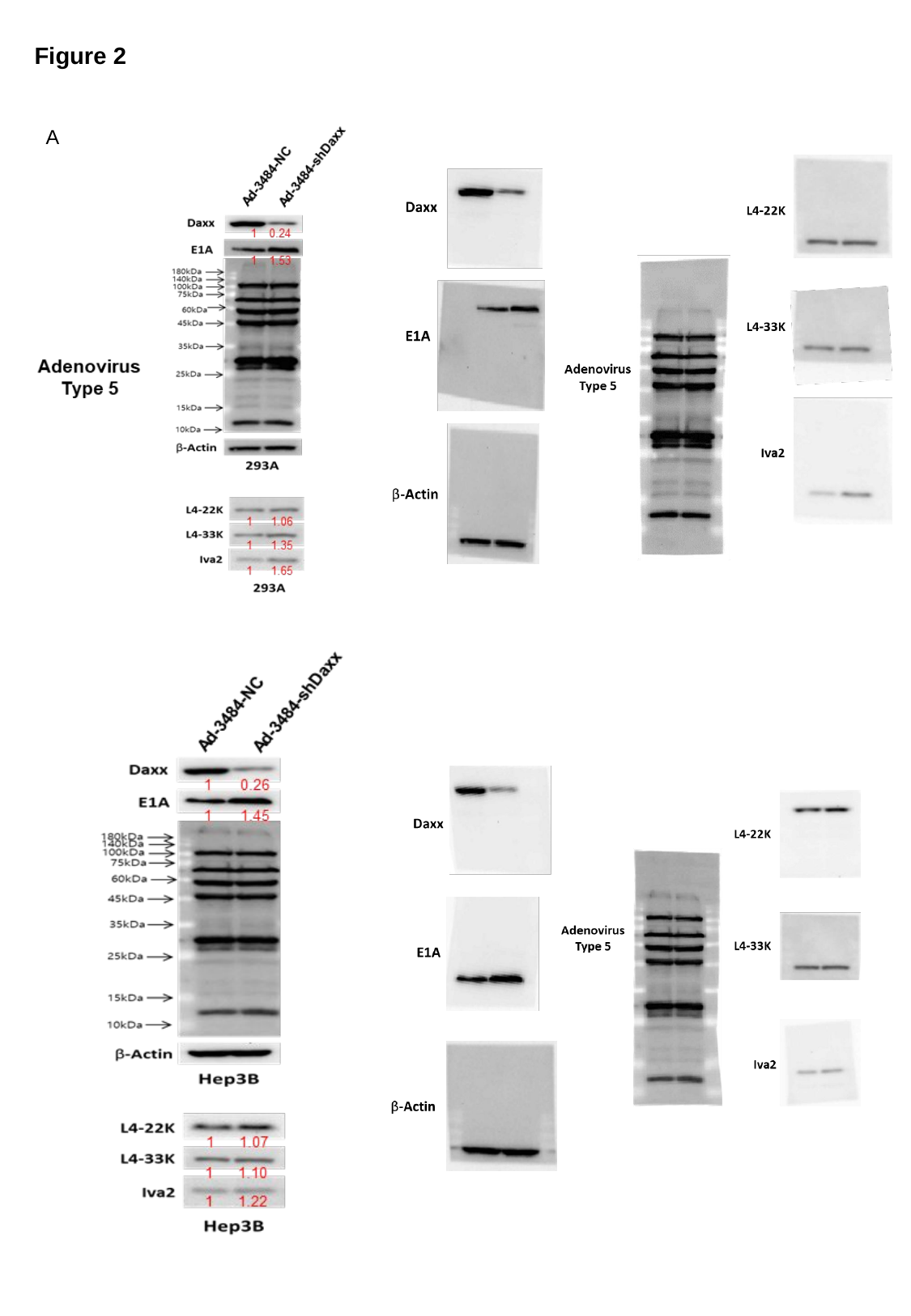

Figure 2
A

## Slide 3
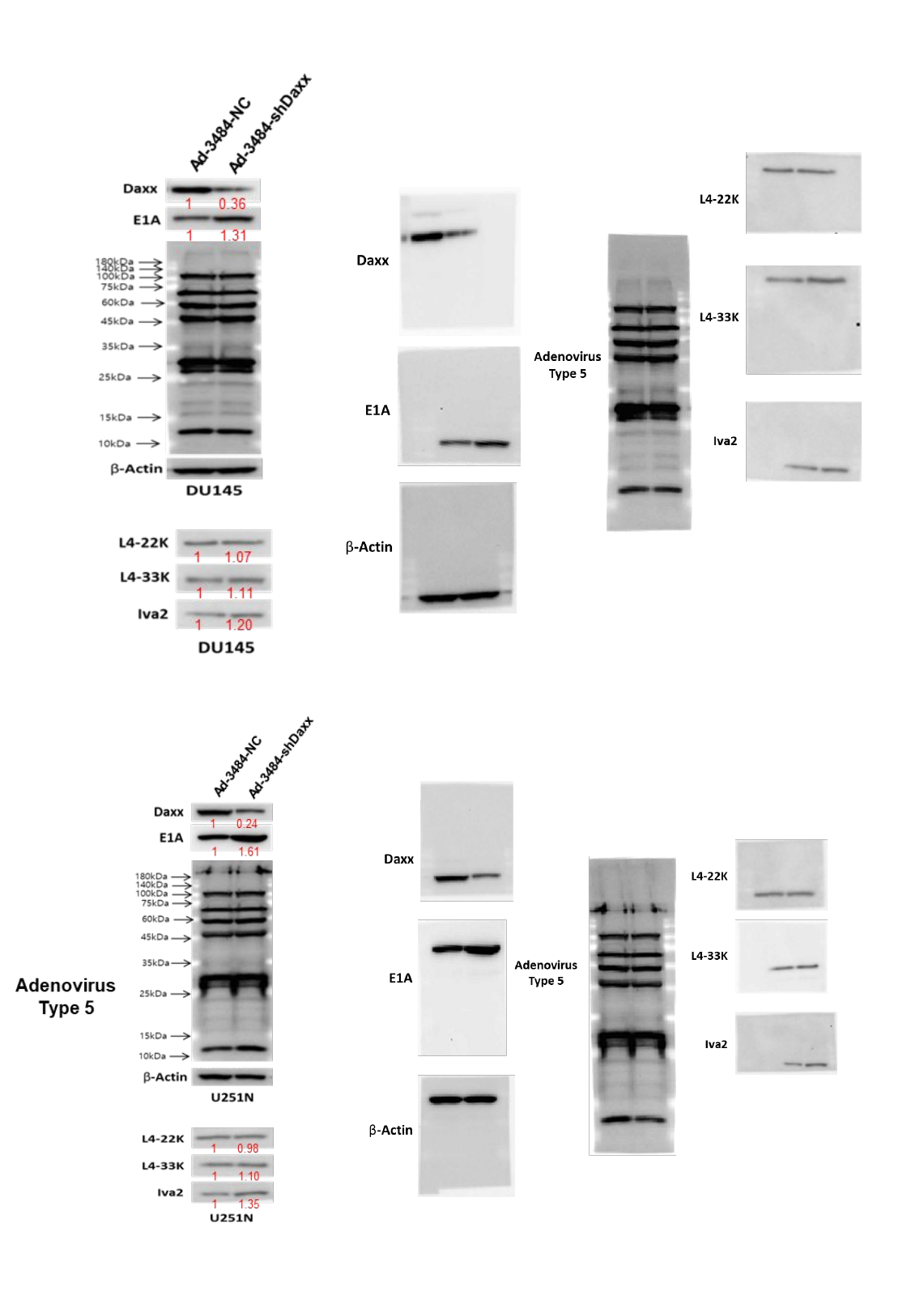

## Slide 4
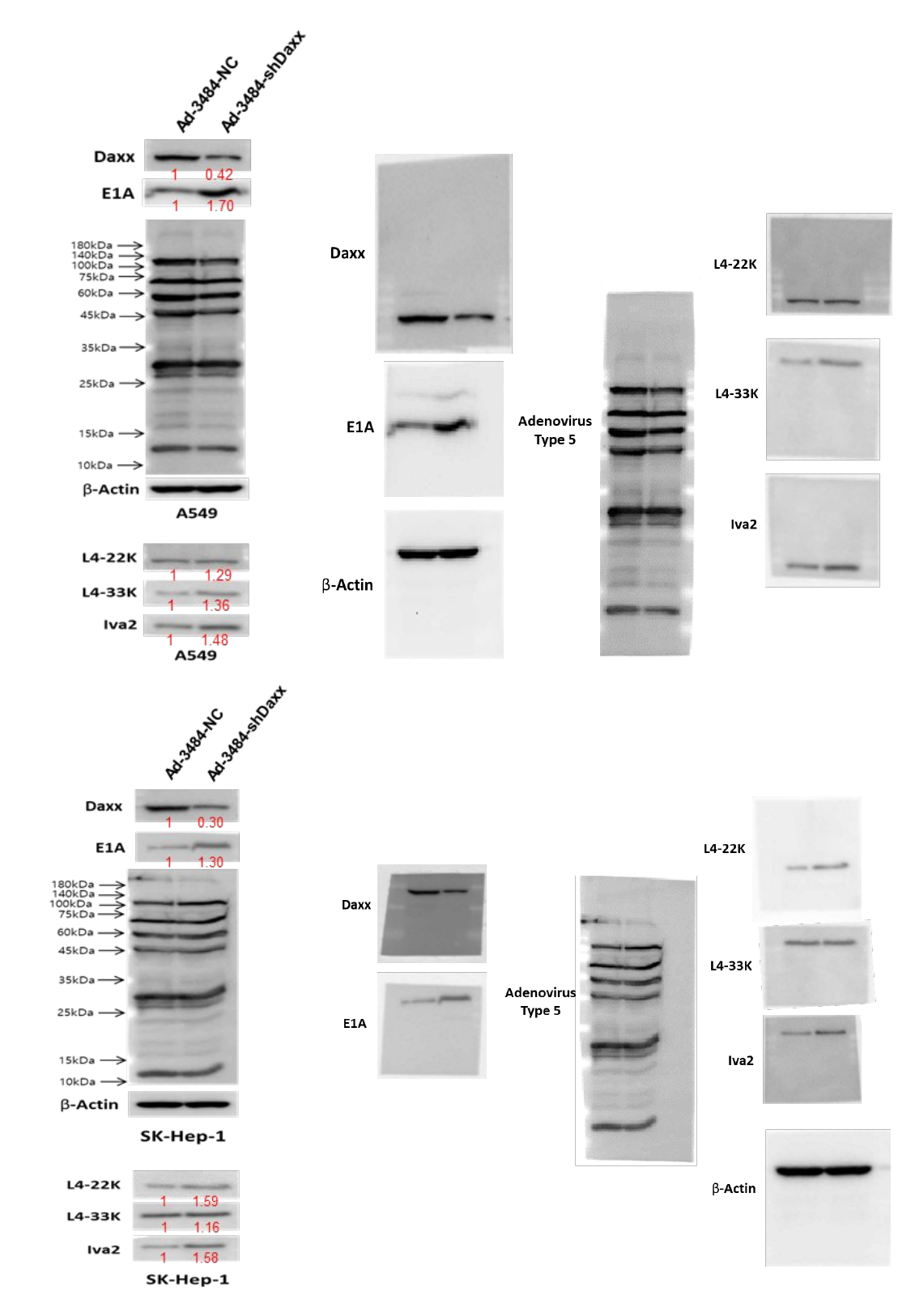

## Slide 5
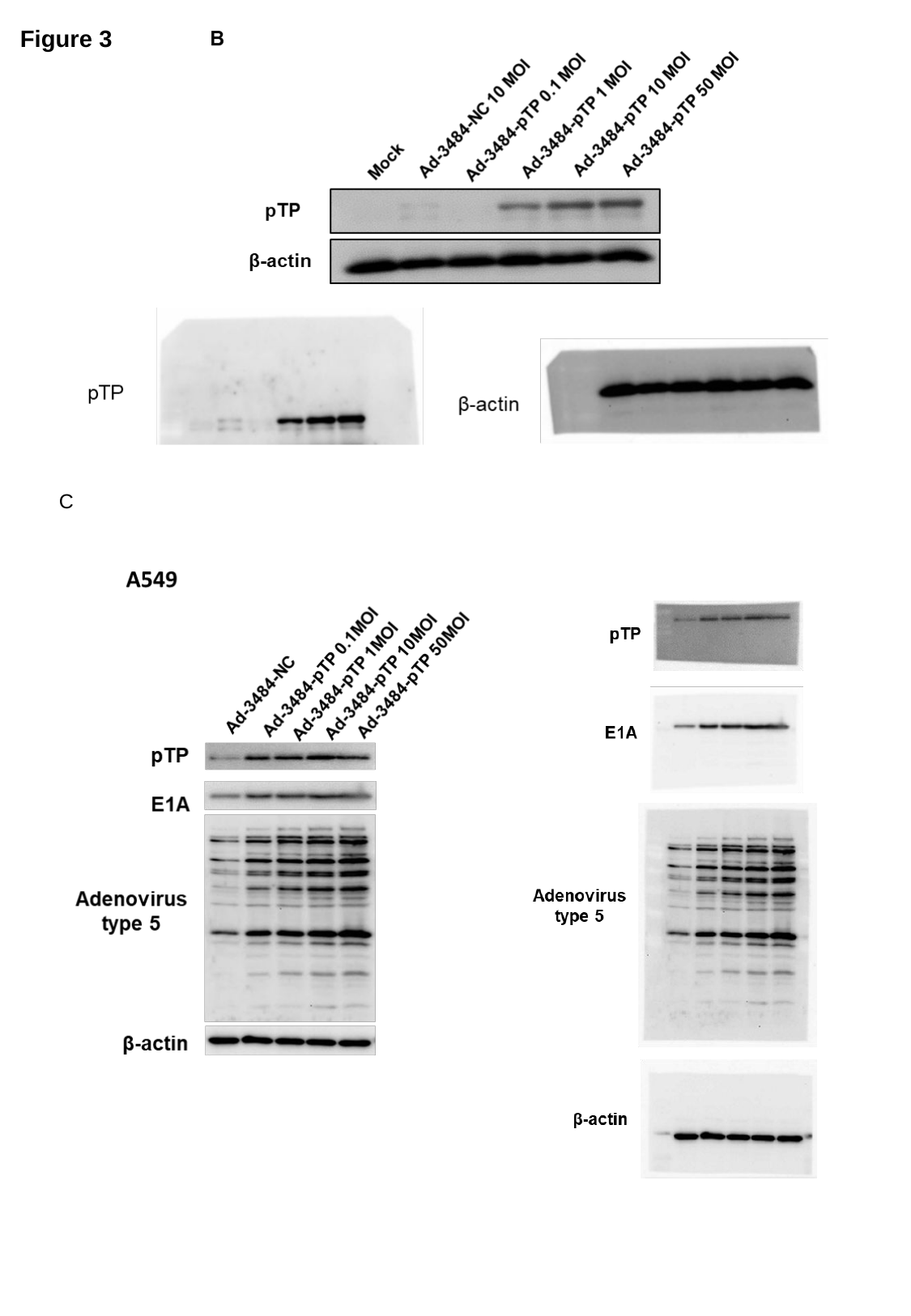

Figure 3
C

## Slide 6
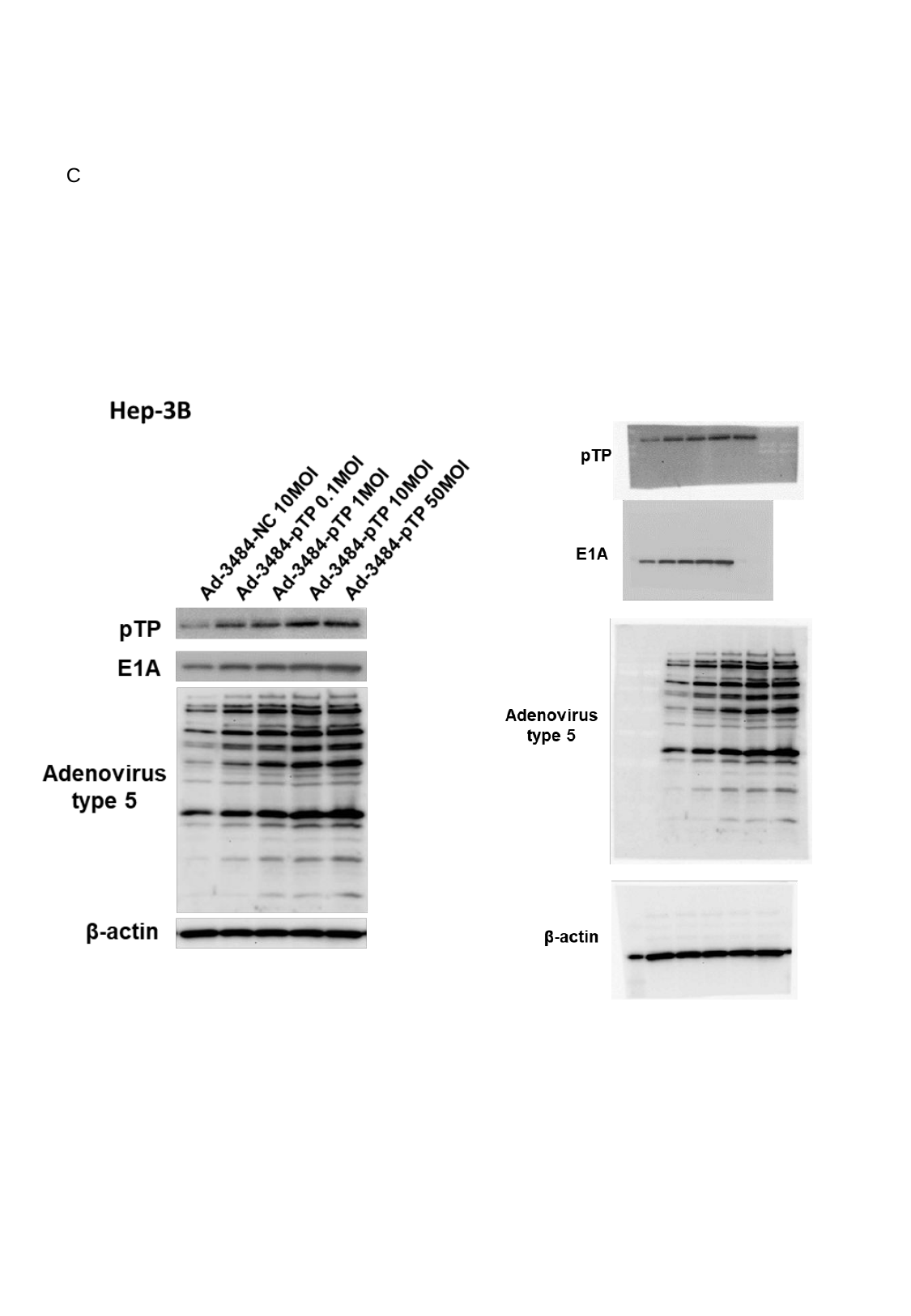

C

## Slide 7
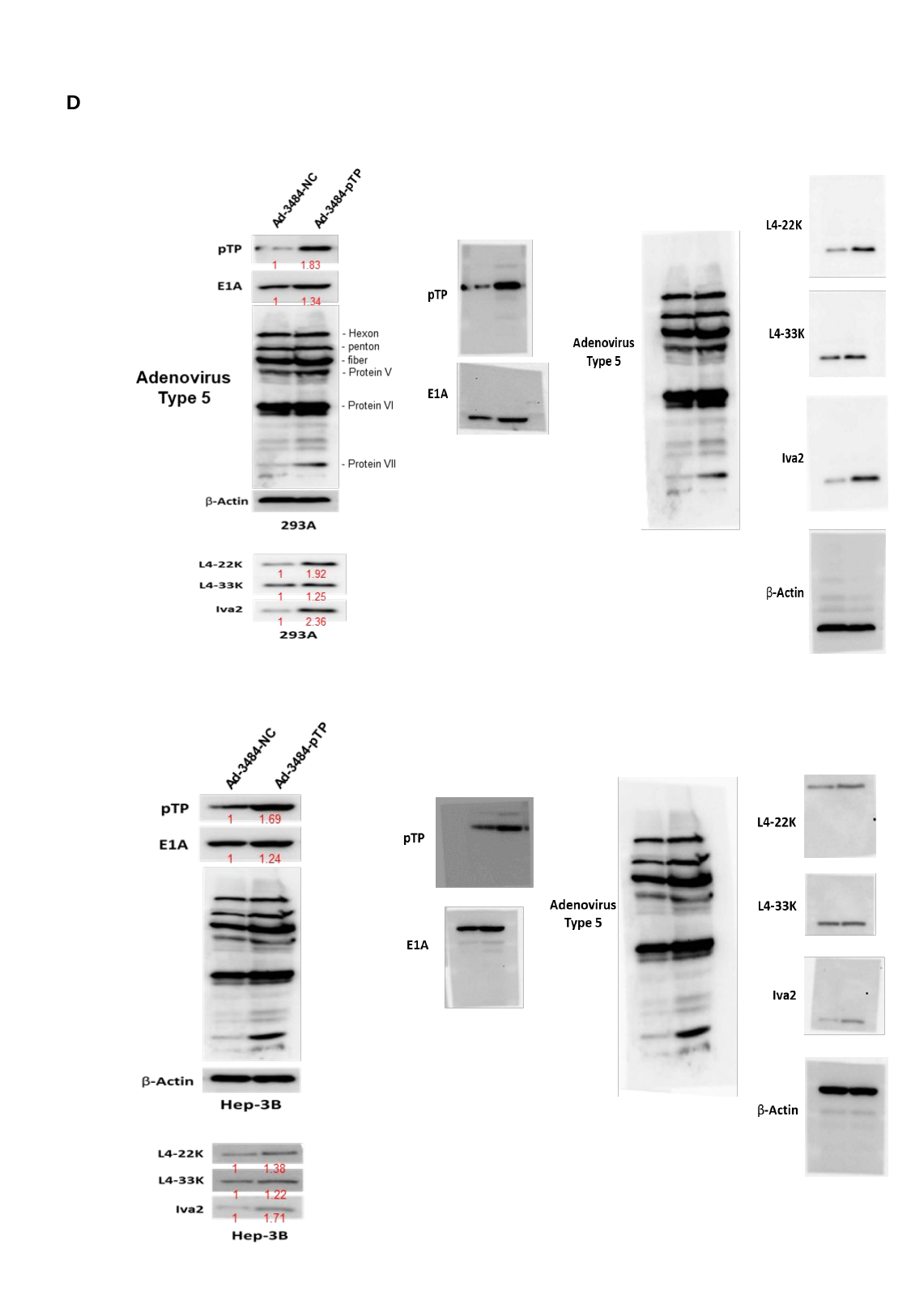

D

## Slide 8
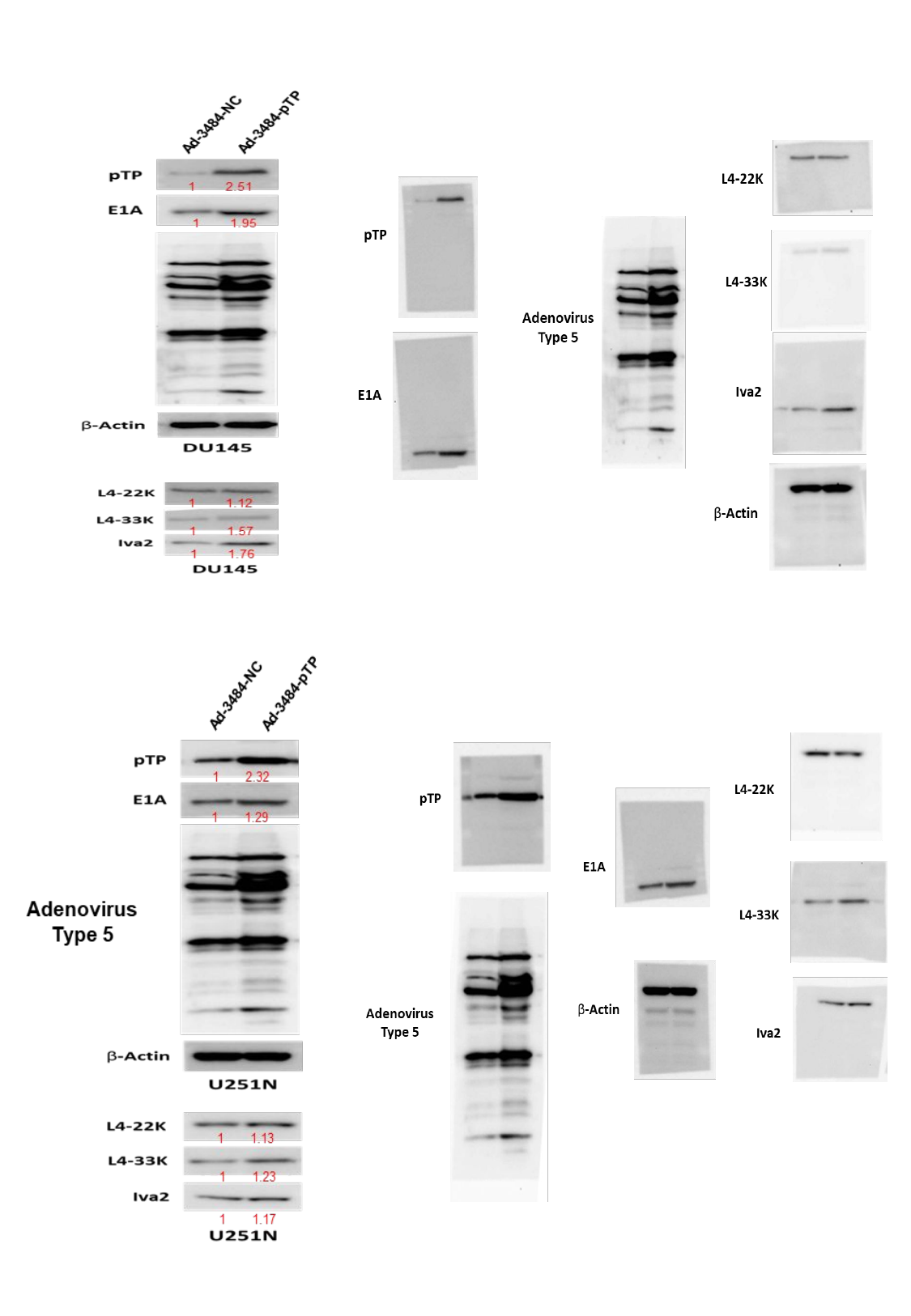

## Slide 9
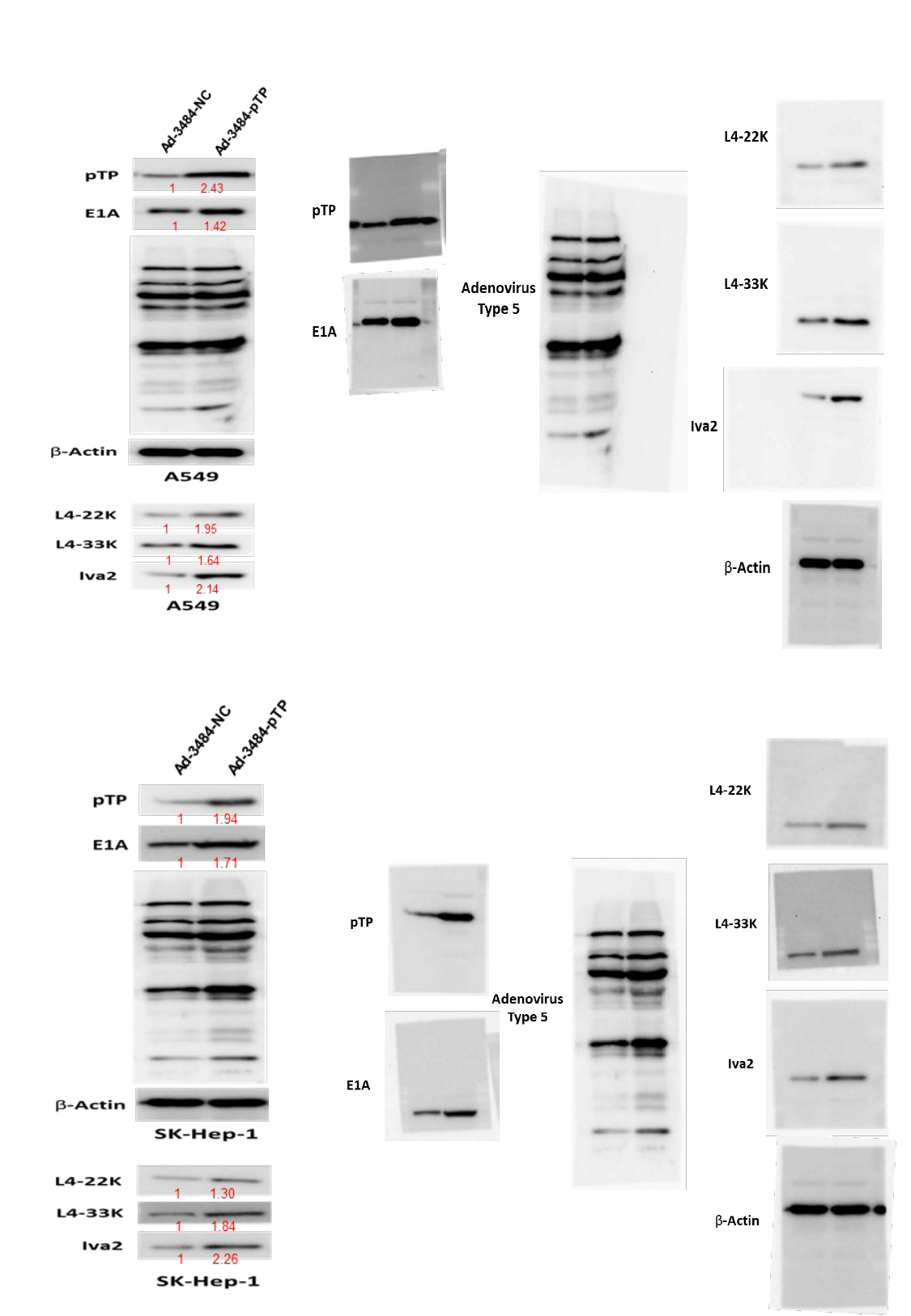

## Slide 10
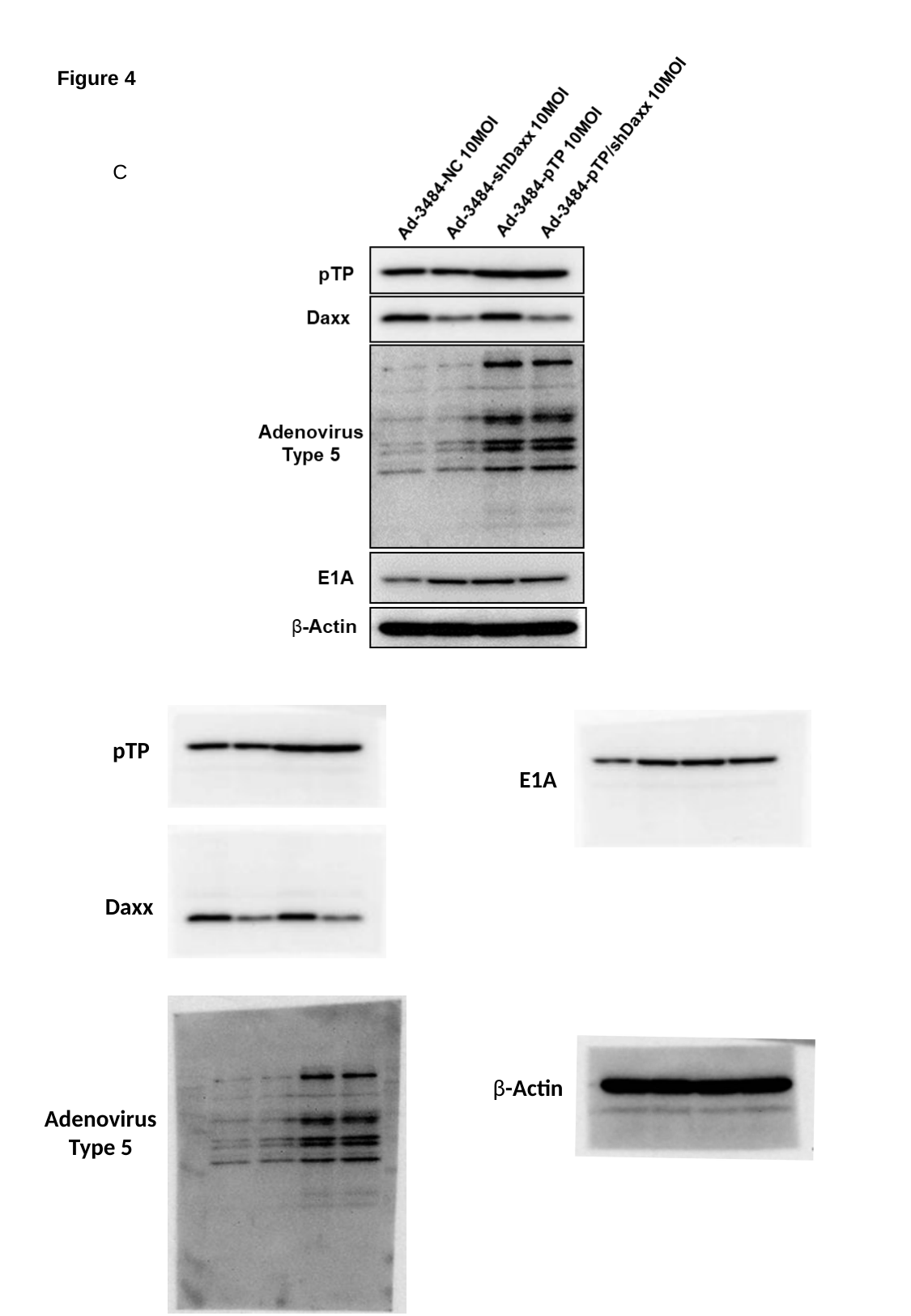

Figure 4
C
pTP
E1A
Daxx
β-Actin
Adenovirus Type 5

## Slide 11
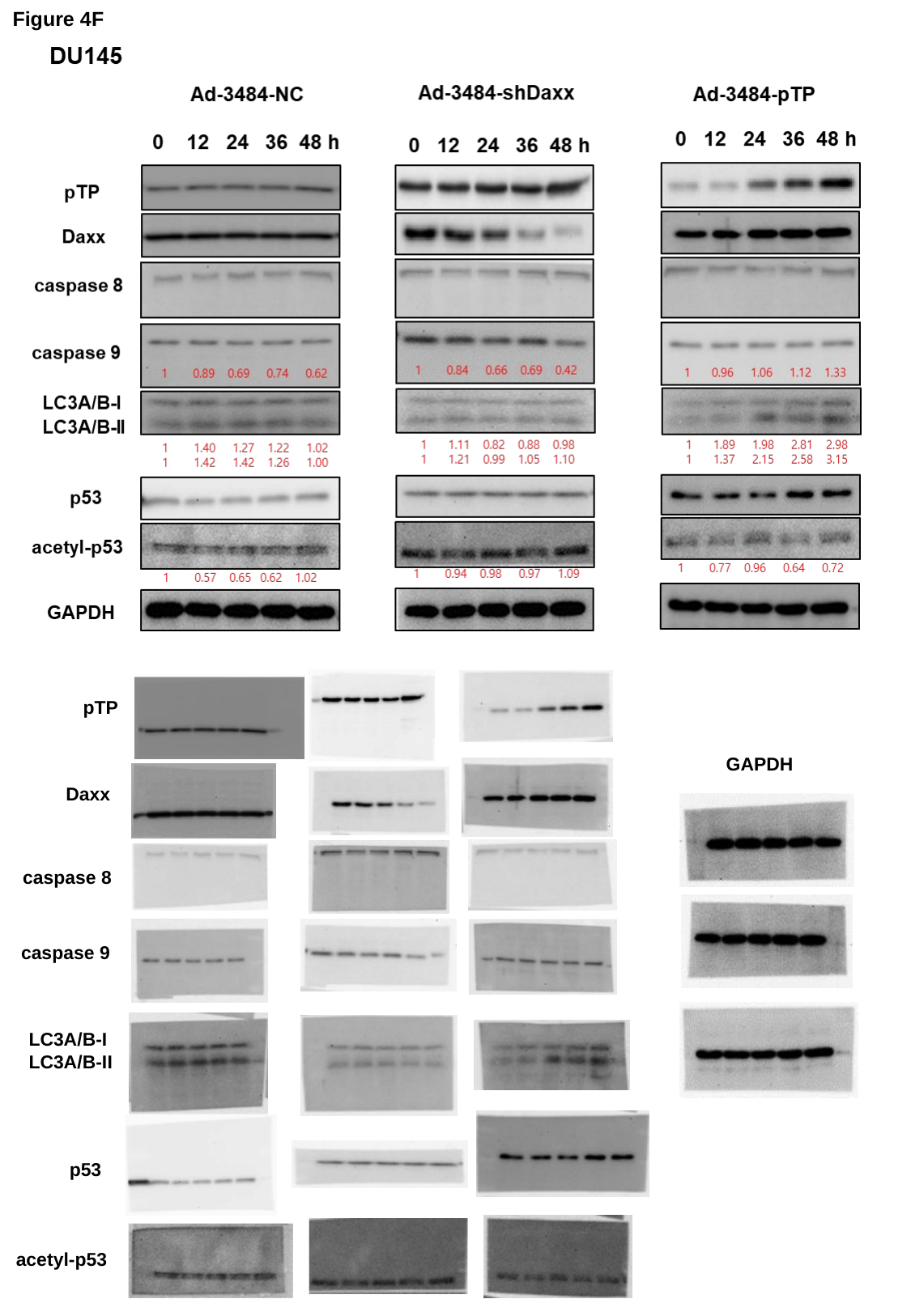

Figure 4F
pTP
GAPDH
Daxx
caspase 8
caspase 9
LC3A/B-I
LC3A/B-II
p53
acetyl-p53

## Slide 12
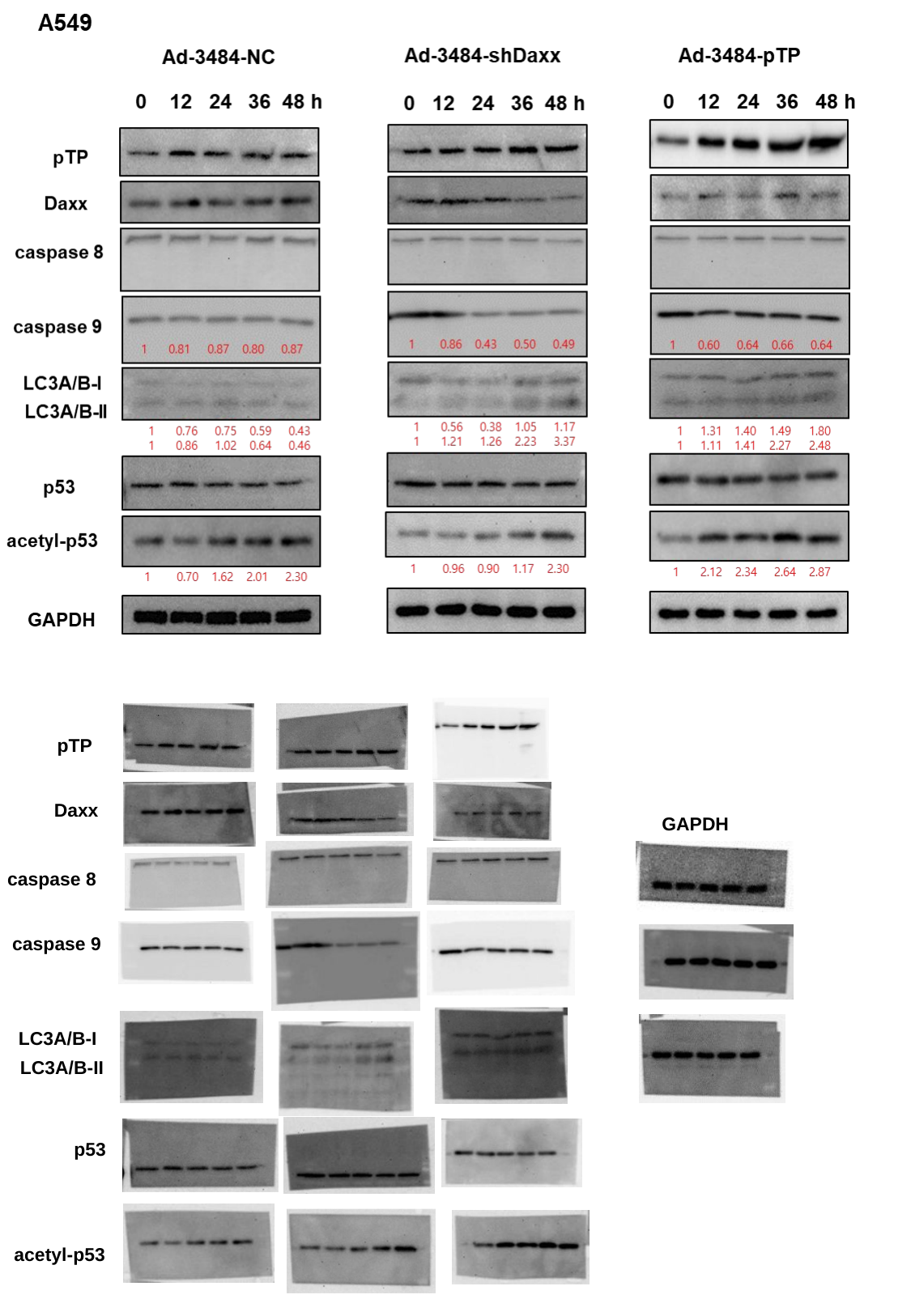

pTP
Daxx
GAPDH
caspase 8
caspase 9
LC3A/B-I
LC3A/B-II
p53
acetyl-p53

## Slide 13
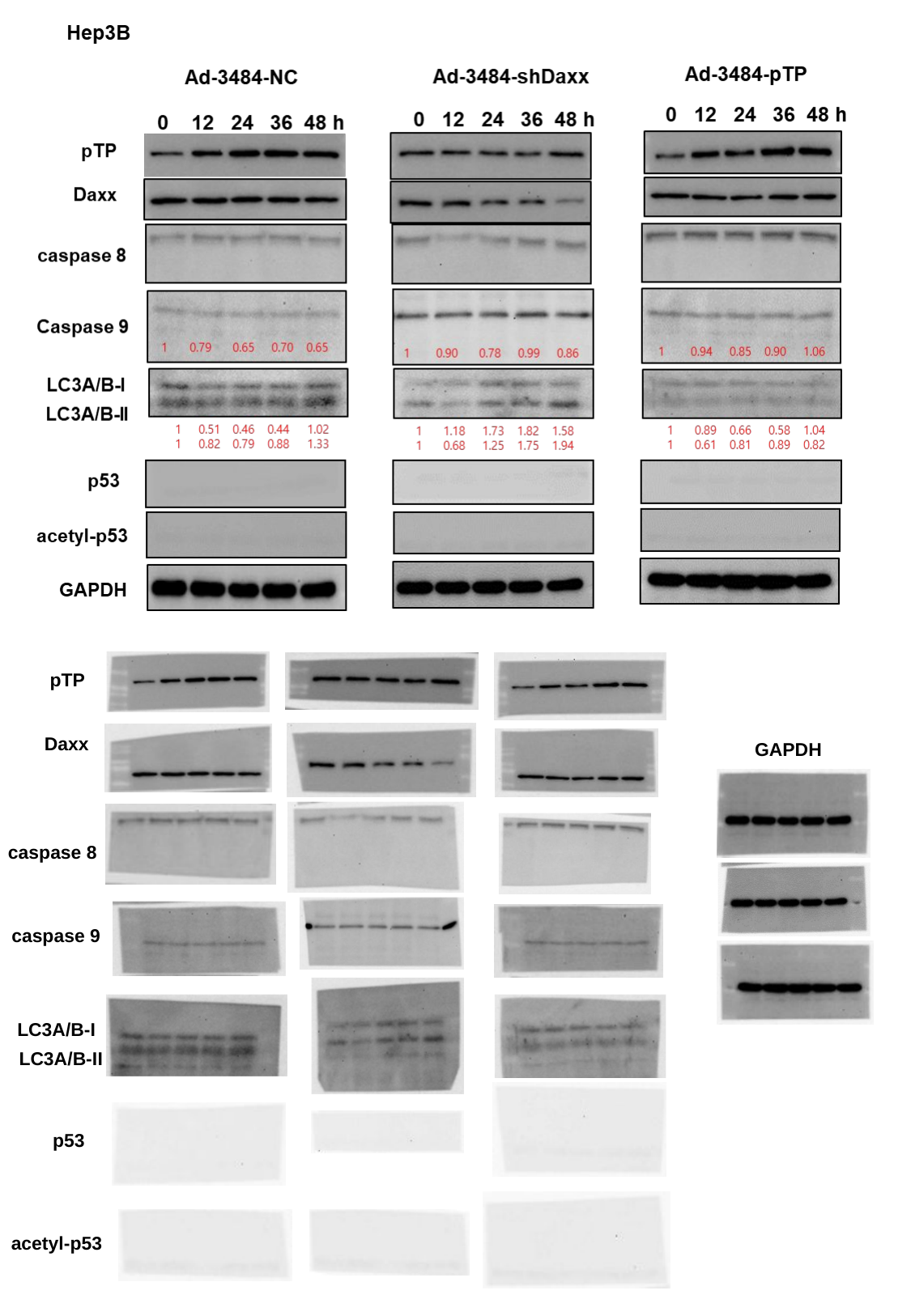

pTP
Daxx
GAPDH
caspase 8
caspase 9
LC3A/B-I
LC3A/B-II
p53
acetyl-p53

## Slide 14
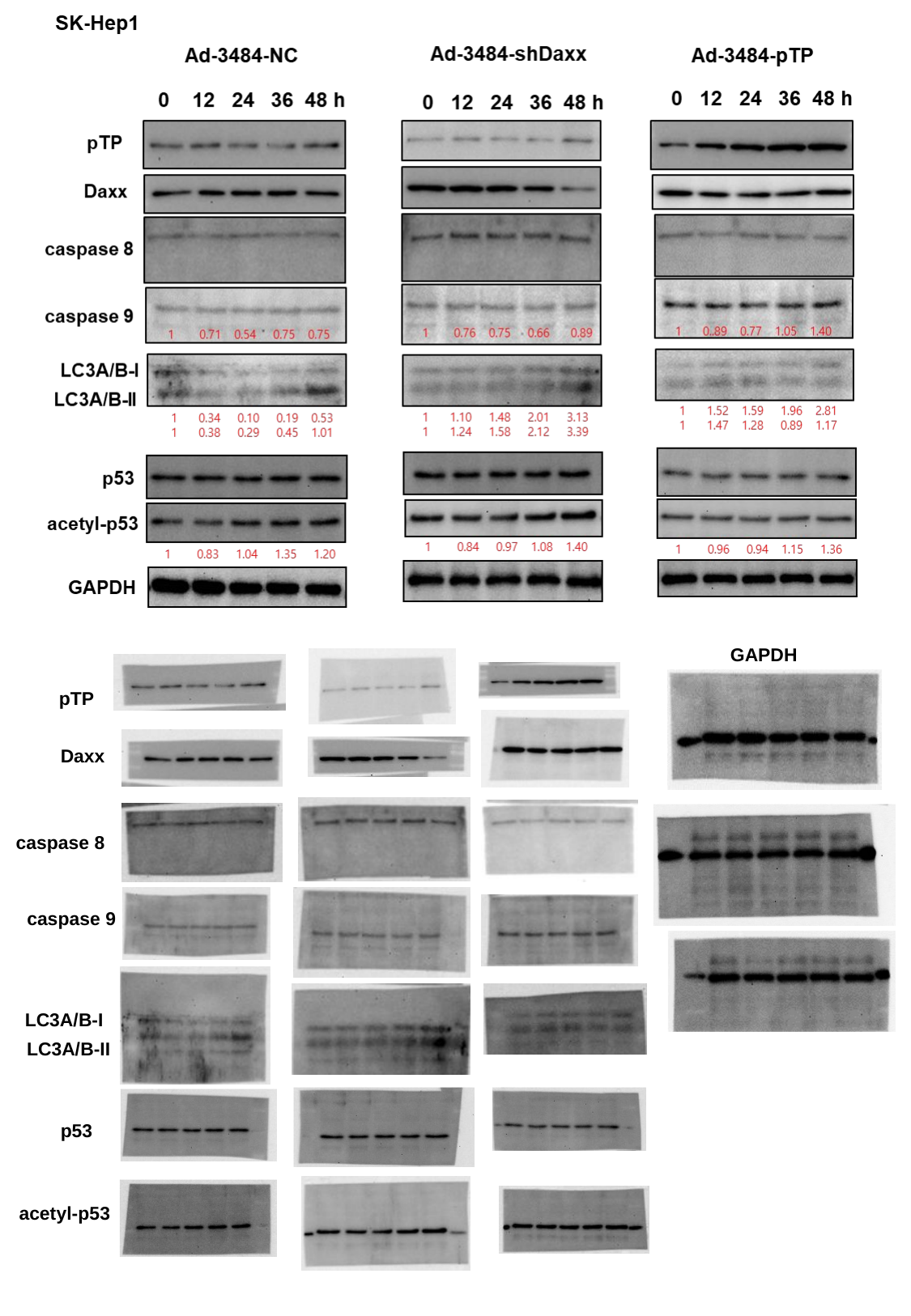

GAPDH
pTP
Daxx
caspase 8
caspase 9
LC3A/B-I
LC3A/B-II
p53
acetyl-p53
